# Sex-specific associations of adolescent motherhood with cognitive function, behavioral problems, and autistic-like traits in offspring and the mediating roles of family conflict and altered brain structure

**DOI:** 10.1186/s12916-024-03442-8

**Published:** 2024-06-05

**Authors:** Tai Ren, Lingli Zhang, Yongjie Liu, Qingli Zhang, Yunjun Sun, Wei Zhou, Like Huang, Ming Wang, Yiwei Pu, Runqi Huang, Jingyu Chen, Hua He, Tailin Zhu, Susu Wang, Weiran Chen, Qianlong Zhang, Wenchong Du, Qiang Luo, Fei Li

**Affiliations:** 1 Ministry of Education - Shanghai Key Laboratory of Children’s Environmental Health & Department of Developmental and Behavioural Paediatric & Child Primary Care, Xinhua Hospital Affiliated to Shanghai Jiao Tong University School of Medicine, 1665 Kongjiang Road, Shanghai, 200092 China; 2 NTU Psychology, School of Social Sciences, Nottingham Trent University, 50 Shakespeare Street, Nottingham, NG1 4FQ UK; 3 Institute of Science and Technology for Brain-Inspired Intelligence, Ministry of Education-Key Laboratory of Computational Neuroscience and Brain-Inspired Intelligence, Fudan University, 220 Handan Road, Shanghai, 200433 China

**Keywords:** Adolescent pregnancy, Neurodevelopment, Family environment, Sex difference, Brain structure

## Abstract

**Background:**

Previous studies have linked adolescent motherhood to adverse neurodevelopmental outcomes in offspring, yet the sex-specific effect and underlying mechanisms remain unclear.

**Methods:**

This study included 6952 children aged 9–11 from the Adolescent Brain Cognitive Development study. The exposed group consisted of children of mothers < 20 years at the time of birth, while the unexposed group was composed of children of mothers aged 20–35 at birth. We employed a generalized linear mixed model to investigate the associations of adolescent motherhood with cognitive, behavioral, and autistic-like traits in offspring. We applied an inverse-probability-weighted marginal structural model to examine the potential mediating factors including adverse perinatal outcomes, family conflict, and brain structure alterations.

**Results:**

Our results revealed that children of adolescent mothers had significantly lower cognitive scores (β, − 2.11, 95% CI, − 2.90 to − 1.31), increased externalizing problems in male offspring (mean ratio, 1.28, 95% CI, 1.08 to 1.52), and elevated internalizing problems (mean ratio, 1.14, 95% CI, 0.99 to 1.33) and autistic-like traits (mean ratio, 1.22, 95% CI, 1.01 to 1.47) in female. A stressful family environment mediated ~ 70% of the association with internalizing problems in females, ~ 30% with autistic-like traits in females, and ~ 20% with externalizing problems in males. Despite observable brain morphometric changes related to adolescent motherhood, these did not act as mediating factors in our analysis, after adjusting for family environment. No elevated rate of adverse perinatal outcomes was observed in the offspring of adolescent mothers in this study.

**Conclusions:**

Our results reveal distinct sex-specific neurodevelopmental outcomes impacts of being born to adolescent mothers, with a substantial mediating effect of family environment on behavioral outcomes. These findings highlight the importance of developing sex-tailored interventions and support the hypothesis that family environment significantly impacts the neurodevelopmental consequences of adolescent motherhood.

**Supplementary Information:**

The online version contains supplementary material available at 10.1186/s12916-024-03442-8.

## Background

Globally, approximately 12 million adolescents between the ages of 15 and 19 become mothers each year [1, 2]. While adolescent motherhood has been linked to adverse neurodevelopmental outcomes in offspring [3–5], the underlying mechanisms of these associations are not yet fully understood. Prior research has often limited its focus to single pathways, such as adverse birth outcomes [6], suboptimal family environment [7, 8], or altered brain structures [9], making it challenging to disentangle the contributions of each factor. Understanding the underlying mechanism is crucial for guiding health initiatives to reduce potentially preventable adverse neurodevelopmental outcomes in the children of adolescent mothers.

Previous studies have established an association between adolescent motherhood and various adverse neurodevelopmental outcomes in offspring throughout childhood and adolescence. These outcomes encompass impaired cognitive ability [5], increased emotional and behavioral problems [9, 10], reduced social competence [5], higher autistic-like traits [11], and an elevated risk of mental illness [3]. These associations could vary by sex, given the well-documented disparities in brain development that can result in varying susceptibility to both prenatal and postnatal neurodevelopmental risk factors [12], such as prenatal stress [13] and the parent–child environment [14]. However, empirical studies examining the sex-specific effects of adolescent motherhood on offspring neurodevelopmental outcomes are scarce [10, 15]. In this study, we investigated the sex-specific associations of adolescent motherhood with three key neurodevelopmental outcomes in offspring: cognitive function, autistic-like traits, and emotional and behavioral problems. Regarding emotional and behavioral problems, we were interested in two main dimensions: internalizing (representing conditions characterized by anxiety, depressive, and somatic symptoms) and externalizing problems (representing conditions characterized by impulsive, disruptive conduct, and substance use symptoms) [16, 17].

We draw on data from a large nationwide sample of children from the Adolescent Brain Cognitive Development (ABCD) study. Furthermore, we assessed the mediating effects of adverse birth outcomes and family environment on these associations and examined differences in brain structures that might be attributed to adolescent motherhood, adjusting for family environment.

## Methods

### Study design

The ABCD study recruited over 11,000 children aged 9 to 11 years from 21 centers across the USA, generally reflecting the diversity of the national adolescent population [18]. The ABCD investigators obtained parents’ full written informed consent and children’s assent. More details of the study protocols are provided at the ABCD website (https://abcdstudy.org/scientists/protocols/) and are described elsewhere [18, 19]. The dataset used for this investigation was from the Annual Curated Data Release 5.0 from the ABCD consortium (https://abcdstudy.org/index.html).

From the initial pool of 11,868 participants, we excluded children with no records of maternal or paternal age (*N* = 706) and those whose parents were older than 35 years of age at the time of their birth (*N* = 4210). The exposed group was defined as children born to mothers under 20 years of age, while the unexposed group was defined as children born to mothers aged between 20 and 35 years. Due to an insufficient sample size of children born to only adolescent fathers (*N* = 63), a 2 × 2 factorial design (adolescent motherhood × adolescent fatherhood) was not feasible.

### Maternal age, outcome measures, and family environment

Maternal age at childbirth was retrospectively collected in a structured interview at recruitment. The corresponding question was “How old were you/biological mother when the child was born?”. Maternal age younger than 20 years at childbirth was defined as adolescent pregnancy according to the World Health Organization’s definition of adolescent pregnancy [1]. Maternal age was additionally analyzed as a continuous variable for comparison.

Cognitive function was assessed with the NIH Toolbox cognition measures, a widely used indicator [20]. NIH Toolbox consists of seven tasks and generates three composite scores: a total score, a crystalized intelligence score, and a fluid intelligence score. A lower score indicates worse cognitive function.

Autistic-like traits was measured by the Short-Social Responsiveness Scale (SSRS), which is a shortened version (11-item) of the 65-item Social Responsiveness Scale, and has been extensively validated [21]. In the ABCD study, the SSRS showed an area under curve of 0.92 in predicting parent-reported diagnosis of autism spectrum disorders. Items in the SSRS were rated by a parent where a higher total score indicates greater severity of social impairment and restricted repetitive behaviors.

Emotional and behavioral problems were assessed with the Child Behavioral Checklist (CBCL). CBCL contains 113 caregiver-reported items that measure a broad scope of emotional and behavioral problems [22]. Our main analysis focused only on the total score and the externalizing and internalizing syndrome scales due to insufficient sample size, though in exploratory analyses we investigated other subdomains of CBCL, including eight syndrome scales, six DSM-oriented scales, and three syndrome scales updated in 2007.

The family environment was measured by multiple indicators following the methodology of Thijssen et al. [23]. These indicators comprised the child-reported abbreviated version of the maternal acceptance scale of the Child Report of Parent Behavior Inventory, the conflict scale from the Family Environment Scale, the Parental Monitoring Survey, the parent-reported conflict scale from the Family Environment Scale, and one single background question from the Kiddie Schedule for Affective Disorders and Schizophrenia (i.e., “in general, how do you and your child get along?”), parental segregation, and parental psychopathology measured by the Achenbach System of Empirically Based Assessment Adult Self-Report. We assessed the family environment in the same procedure as reported previously [23] while excluding items that were not considered as mediators in this study. These excluded variables included planned/unplanned pregnancy, parental education, and parental income. Parental education and parental income were considered as confounders in our analysis (Additional file 1: Fig. S1). As indicated in the previous study [23], a lower family environment score indicates a more stressful environment.

### Covariates and neuroimage data

Potential confounders and mediators were selected based on directed acyclic graphs depicting the best-known relations between the variables in this study (Additional file 1: Fig. S2). Confounders included paternal age at birth (< 20 years, or 20–35 years), parental annual income level (range, 1 to 10; see Additional file 1: Table S1 for specific coding), and race/ethnicity (White, Black, Hispanic, or other). We also adjusted for the sex of the offspring, and their age at outcome assessment. Parental annual income level was described as a trichotomized variable (< $50,000, $50,000–100,000, ≥ $100,000) while adjusted for as a continuous variable. Notably, parental income was considered a proxy for socioeconomic status both prior to and subsequent to pregnancy—the former being a confounder and the latter a potential mediator. As such, we compared two analytical models: one adjusted for parental income to appraise its confounding effect and another unadjusted to assess mediation. Parental education was not included in the analysis due to a high rate of missing data (~ 20%) and a substantial correlation with parental income [24].

Mediators evaluated in the study included the family environment [7] (as detailed previously), adverse perinatal outcomes [25, 26], and structural change of the brain [9]. Perinatal outcomes included self-reported gestational hypertension (yes, or no), gestational diabetes mellitus (yes, or no), preterm birth (yes, or no), and low birth weight (yes, or no). We obtained the structural magnetic resonance imaging data (MRI, T1 image; *N* = 6870) preprocessed and quality-controlled by the ABCD team [19]. We excluded poor quality T1 scans (*N* = 3), FreeSurfer outputs not passing manual QC (*N* = 270), and any incidental findings noted from neuroradiological read of the structural MRI images (*N* = 293) [27]. Herein, we focused on an ABCD summary of standard FreeSurfer v5.3.0 data from the Destrieux atlas regions of interest, including the cortical area, volume, and thickness of 68 cortical regions and 40 subcortical regions [28].

### Statistical analyses

Demographic characteristics were compared with t-test and chi-squared for continuous and categorical variables, respectively, stratified by sex. A generalized linear mixed-effect model was applied to investigate the associations of adolescent motherhood with outcomes including neurocognitive outcomes, family environment, and brain morphometrics. The mixed models were specified as family nested within site, in order to account for the data structure of the ABCD study [29]. For normally distributed outcomes including NIH Toolbox, family environment, and brain morphometrics, we fitted linear models to estimate βs with 95% confidence intervals (CIs). For right-skewed outcomes, including CBCL and SSRS, we fitted Poisson regression, after confirming the overdispersion test, to estimate mean ratios with 95% confidence intervals (for details of model selection, see Additional file 1: Additional Methods S1). Potential confounders were adjusted for as described in the covariates section. In addition, when investigating structural MRI, we additionally adjusted for T1 image signal-to-noise and intracranial volume (for analyses on cortical thickness, the intracranial volume was not adjusted for). We tested the interaction term of adolescent motherhood × sex and stratified the analyses by sex when the interaction was significant. To account for multiple comparison, we calculated the false discovery rate (FDR) for analyses on MRI measures. Missing values were imputed by chained equations with package mice (version 3.15.0).

The family environment scores were calculated using structural equation modeling by the package lavaan (version 0.6–16) [30]. Mediation analyses were performed to investigate the mediation roles of adverse perinatal outcomes, family environment, and brain structure in the observed associations. We applied a counterfactual framework with inverse probability of treatment weighting methods, as described previously [31]. The proportion eliminated (PE) was calculated to measure the reduction in total effect attributable to the mediator. Notably, no elevated rate of adverse perinatal outcomes was observed in the offspring of adolescent mothers, even after considering potential bias from higher data omission in the exposed. To account for the cohort effect, we additionally adjusted for birth year (as a categorical variable) in a supplementary analysis. To test the robustness of findings in neuroimage, we randomly divided the study population into two groups based on study sites (11 centers in group 1 and the remaining in group 2). We also analyzed the MRI data at the 2-year follow-up. We also reported potential confounding due to unmeasured confounders by computing the e-values in observed associations. The e-value represents the minimum magnitude of association on the risk ratio scale that an unobserved confounding factor would require with both the treatment and the outcome to completely account for a specific treatment-outcome association, while considering the measured covariates. We did not observe higher rates of adverse perinatal outcomes in the adolescent mothers; thus, mediation analyses for adverse perinatal outcomes were not pursued. R version 4.1.2 (R Foundation for Statistical Computing, Vienna, Austria) was used for the analyses.

## Results

Of the 6952 (52.0% male) eligible children, 6302 (90.7%) had mothers who were within the age range of 20 to 34 years at the time of birth. A total of 650 (9.3%) children had mothers younger than 20 years at birth. Children of both sexes born to adolescent mothers were more likely to have a younger father, to have lower parental educational and income levels, and to be members of racial and ethnic minority groups (Table 1). The mean age at assessing the cognitive, behavioral, and autistic-like outcomes was comparable between the two groups of both sexes (mean [standard deviation, SD], 9.9 [0.6], for cognition and behavioral problem assessment; mean [SD], 10.9 [0.6], for autistic-like traits).

**Table 1:** Baseline characteristics of involved children from the Adolescent Brain Cognitive Development study

	**Male**			**Female**		
	**Mother aged 20–35 years**	**Mother aged < 20 years**	***P* value**	**Mother aged 20–35 years**	**Mother aged < 20 years**	***P* value**
	**(*N* = 3297)**	**(*N* = 321)**		**(*N* = 3005)**	**(*N* = 329)**	
**Paternal age at childbirth, years**			< 0.001			< 0.001
< 20	29 (0.9)	155 (48.3)		34 (1.1)	148 (45.0)	
20–35	3268 (99.1)	166 (51.7)		2971 (98.9)	181 (55.0)	
**Race/Ethnicity (%)**			< 0.001			< 0.001
White	1788 (54.2)	62 (19.3)		1581 (52.6)	62 (18.8)	
Black	457 (13.9)	123 (38.3)		470 (15.6)	113 (34.3)	
Hispanic	678 (20.6)	105 (32.7)		611 (20.3)	101 (30.7)	
Other	361 (10.9)	30 (9.3)		334 (11.1)	49 (14.9)	
Missing	13 (0.4)	1 (0.3)		9 (0.3)	4 (1.2)	
**Parental annual income level, (%)**			< 0.001			< 0.001
< $50,000	984 (29.8)	215 (67.0)		937 (31.2)	212 (64.4)	
$50,000–100,000	1035 (31.4)	82 (25.5)		948 (31.5)	84 (25.5)	
≥ $100,000	1278 (38.8)	24 (7.5)		1120 (37.3)	33 (10.0)	
Missing	258 (7.8)	51 (15.9)		230 (7.7)	58 (17.6)	
**Parental average educational level, years, mean (SD)**	15.5 (2.1)	13.6 (1.6)	< 0.001	15.5 (2.1)	13.4 (1.9)	< 0.001
Missing (%)	625 (19.0)	115 (35.8)		595 (19.8)	122 (37.1)	
**Age at assessing cognitive and behavioral problems, years, mean (SD)**	9.9 (0.6)	9.9 (0.6)	0.97	9.9 (0.6)	9.9 (0.6)	0.63
**Age at assessing autistic-like traits, years, mean (SD)**	10.9 (0.7)	10.9 (0.6)	0.60	10.9 (0.6)	10.9 (0.7)	0.87
Missing (%)	160 (4.9)	35 (10.9)		173 (5.8)	30 (9.1)	
**Birth year (%)**			0.002			0.03
2005/2006^a^	428 (13.0)	30 (9.3)		324 (10.8)	35 (10.6)	
2007	1155 (35.0)	93 (29.0)		1068 (35.5)	91 (27.7)	
2008	1196 (36.3)	150 (46.7)		1100 (36.6)	142 (43.2)	
2009	518 (15.7)	48 (15.0)		513 (17.1)	61 (18.5)	
**Family environment**						
** Family environment score, mean (SD)**^**b**^	− 0.035 (0.52)	− 0.14 (0.56)	< 0.001	0.019 (0.50)	− 0.16 (0.60)	< 0.001
**Child-reported**						
** Parental monitoring, mean (SD)**	4.3 (0.5)	4.2 (0.6)	0.066	4.5 (0.5)	4.4 (0.5)	0.12
Missing (%)	5 (0.2)	1 (0.3)		4 (0.1)	0 (0)	
** Parental acceptance, mean (SD)**	2.7 (0.4)	2.7 (0.4)	0.10	2.7 (0.4)	2.7 (0.5)	0.075
Missing (%)	261 (7.9)	31 (9.7)		193 (6.4)	42 (12.8)	
** Family conflict, mean (SD)**	2.2 (2.0)	2.4 (2.0)	0.082	2.0 (2.0)	2.1 (1.9)	0.21
Missing (%)	7 (0.2)	1 (0.3)		5 (0.2)	0 (0)	
**Parent-reported**						
** Family conflict, mean (SD)**	2.6 (2.0)	2.8 (2.0)	0.072	2.4 (1.9)	2.7 (2.1)	0.021
** Getting along, mean (SD)**	1.2 (0.4)	1.2 (0.5)	0.65	1.2 (0.4)	1.3 (0.5)	0.058
Missing (%)	1 (0.0)	0 (0)		0 (0)	0 (0)	
** Parental psychopathology, mean (SD)**	22.1 (18.6)	24.1 (20.1)	0.085	21.5 (17.9)	26.5 (23.0)	< 0.001
Missing (%)	1 (0.0)	0 (0)		0 (0)	0 (0)	
** Parents still together (%)**	2472 (75.0)	161 (50.2)	< 0.001	2198 (73.1)	152 (46.2)	< 0.001
**Perinatal factors**						
** Maternal gestational hypertension** (%)	392 (11.9)	32 (10.0)	0.46	401 (13.3)	45 (13.7)	0.81
Missing (%)	48 (1.5)	15 (4.7)		32 (1.1)	11 (3.3)	
** Maternal gestational diabetes mellitus** (%)	186 (5.6)	4 (1.2)	0.002	184 (6.1)	11 (3.3)	0.066
Missing (%)	43 (1.3)	15 (4.7)		34 (1.1)	11 (3.3)	
** Preterm birth** (%)	578 (17.5)	44 (13.7)	0.10	553 (18.4)	37 (11.2)	0.002
Missing (%)	15 (0.5)	2 (0.6)		13 (0.4)	6 (1.8)	
** Low birth weight** (%)	398 (12.1)	35 (10.9)	0.75	490 (16.3)	47 (14.3)	0.42
Missing (%)	100 (3.0)	20 (6.2)		93 (3.1)	13 (4.0)	

Monitoring score range, 1 to 5. Parental acceptance range, 1 to 3. Family conflict score range, 0 to 9. Getting along score range, 1 to 3. Psychopathology score range, 0 to 154

^a^Due to the small number of children born in 2005 (less than 10), they were combined with the "2005/2006" group

^b^The family environment score was constructed using below-listed variables using structural equation models (detailed in Fig. S1)

**Fig. 1:**
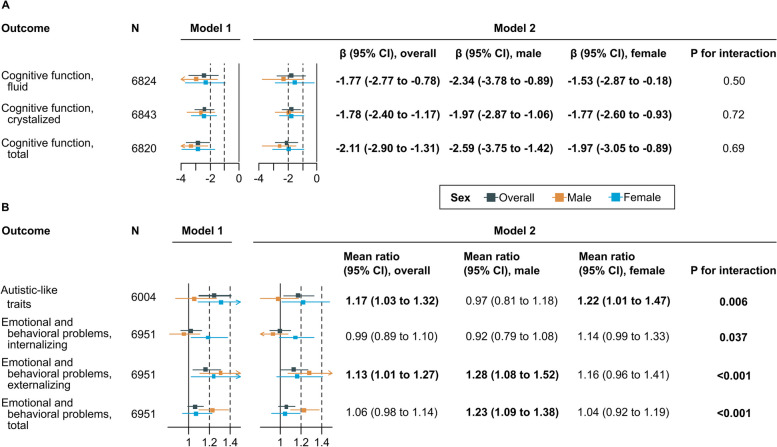
Associations of adolescent motherhood with **A** cognitive function, **B** autistic-like traits, and emotional and behavioral problems, stratified by offspring sex. SSRS, Short-Social Responsiveness Scale; CBCL, Child Behavioral Checklist. Model 1 was fitted adjusted for paternal age less than 20 years at birth, race, sex, and age at outcome assessment. Model 2 was fitted adjusted for paternal age less than 20 years at birth, race, sex, age at outcome assessment, and parental income level. Cognitive function was measured by the NIH Toolbox. Autistic-like traits were measured by the SSRS. Emotional and behavioral problems were measured by the CBCL. Significant findings were enboldened

The average total cognitive scores were 86.3 (SD, 9.0) in the reference children and 80.6 (SD, 9.0) in children who had an adolescent mother at birth, respectively. After adjusting for paternal age of less than 20 years at birth, sex, and race, adolescent motherhood was associated with a worse total cognitive score, measured by NIH Toolbox, in offspring in late childhood (β, − 2.86, 95% CI, − 3.70 to − 2.04; Fig. 1A, model 1). Additionally adjusting for parental income level attenuated this association (β, − 2.11, 95% CI, − 2.90 to − 1.31; Fig. 1A, model 2). In all the following analyses, parental income was adjusted for as a confounder. Specifically, adolescent motherhood was associated with both lower fluid and crystalized intelligence scores (Fig. 1A). The risk estimates were similar in both male and female offspring (Fig. 1A; *P* for interaction, 0.69).

We observed sex-specific associations of adolescent motherhood with autistic-like traits and emotional and behavioral problems. Having a mother aged less than 20 years at birth was associated with a higher mean SSRS score in female offspring by 22% (mean ratio, 1.22, 95% CI, 1.01 to 1.47), but not in male offspring (mean ratio, 0.97, 95% CI, 0.81 to 1.18; *P* for interaction, 0.006; Fig. 1B). Meanwhile, adolescent motherhood was marginally associated with more internalizing problems in female offspring (mean ratio, 1.14, 95% CI, 0.99 to 1.33; *P* for interaction, 0.037; Fig. 1B) and more externalizing problems in male offspring (mean ratio, 1.28, 95% CI, 1.08 to 1.52; *P* for interaction, < 0.001; Fig. 1B). We computed *e*-values for all models with statistically significant associations of adolescent motherhood. The association between adolescent motherhood and internalizing problems in females showed an *e*-value of 1.00 due to its marginal significance. For other outcomes, the *e*-values showed that a minimum risk ratio of 1.11 to 1.57 would be required for an unmeasured confounder to be associated with both the exposure and the outcome to fully explain the observed associations (Additional file 1: Table S2). In additional analyses, treating maternal age as a continuous variable yielded consistent results (Additional file 1: Fig. S3). The results were robust to additional adjustment for the birth year (Additional file 1: Table S3). Exploratory analyses on each task or subdomain of NIH Toolbox and syndrome or DSM-oriented CBCL, respectively, revealed generally consistent patterns with observed associations (Additional file 1: Fig. S4–S5).

**Fig. 2:**
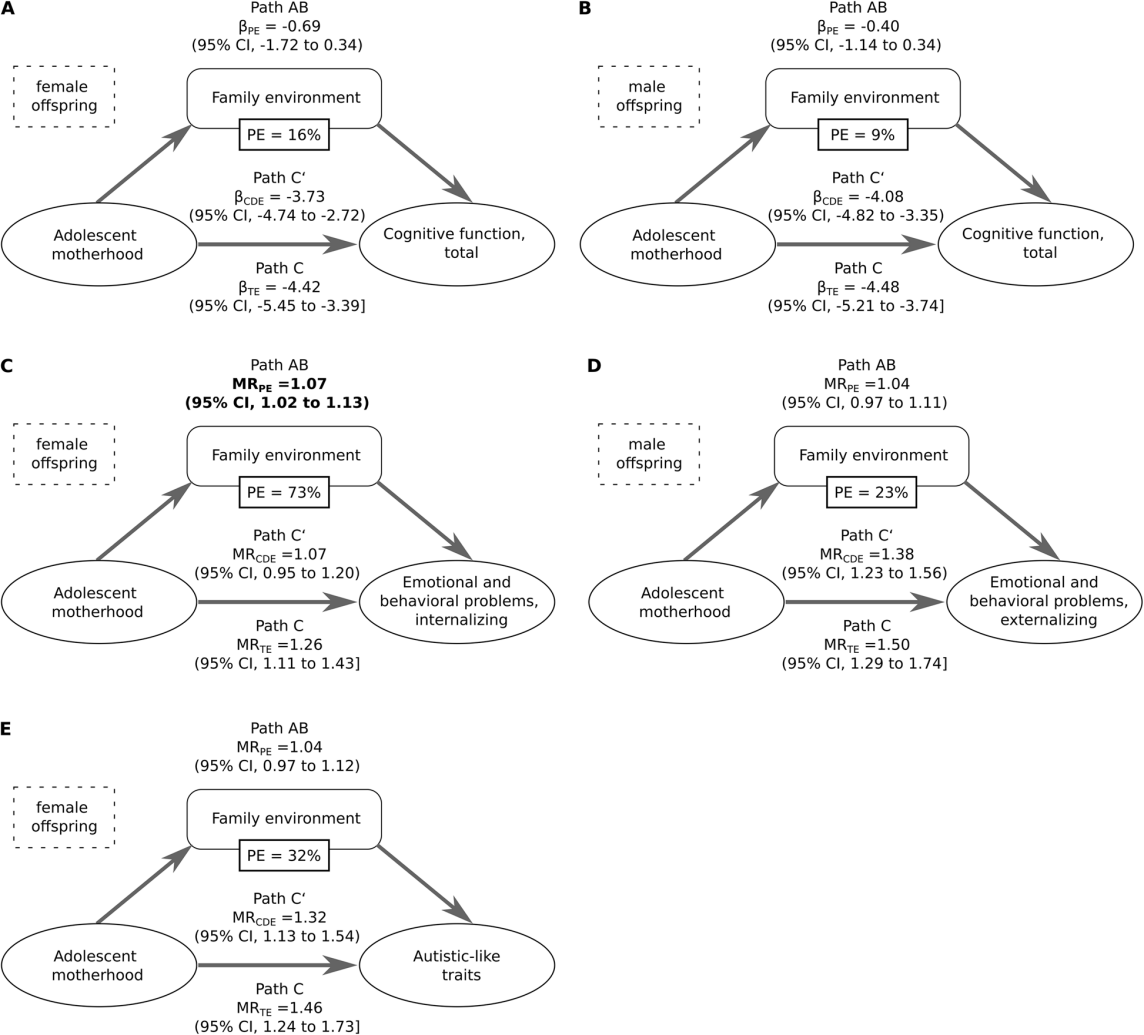
Mediation analysis of family environment in explaining the association of adolescent motherhood with **A** cognitive function in female offspring; **B** cognitive function in male offspring; **C** internalizing problems in female offspring; **D** externalizing problems in male offspring; and **E** autistic-like traits in female offspring. MR, mean ratio; TE, total effect; CDE, controlled direct effect; PE, proportion eliminated. For NIH Toolbox in females (**A**), the indirect path (AB) shows that the family environment score could mediate part of the effect of adolescent motherhood. Path C shows that the risk estimate (β) of adolescent motherhood on the NIH Toolbox was prominent when family environment score was not taken into account. Path C′ indicates the direct effect of adolescent motherhood on NIH Toolbox controlling for the mediator (family environment score). Path C′ shows some reduction in the risk estimate when the effect of family environment score was taken into account. Path AB indicates the extent to which taking the family environment score into account can explain the 16% effect of the adolescent motherhood on NIH Toolbox, though insignificant because the confidence interval of β_PE_ crossed unity. Other mediation analyses were presented in a similar way. Proportion eliminated = (MR_TE_ − MR_CDE_)/(MR_TE_ − 1), or (β_TE_ − β_CDE_)/β_TE_. Proportion eliminated is only presented if the βs of CDE and PE were in the same direction. Cognitive function was measured by the NIH Toolbox. Autistic-like traits were measured by the SSRS. Emotional and behavioral problems were measured by the CBCL. Significant findings on PE were enboldened

In both male and female offspring, adolescent motherhood was associated with a lower family environment score, which indicated a more stressful family environment (Table 1). Children exposed to higher levels of family stress showed lower cognitive function, more internalizing and externalizing problems, and more autistic-like behaviors (Additional file 1: Table S4). We observed that a more stressful family environment mediated more than 70% of the association between adolescent motherhood and internalizing problems in female offspring (PE = 73%, *P* = 0.010; Fig. 2). Additionally, a more stressful family environment mediated approximately 30% in the association with autistic-like traits among female offspring (PE = 32%, *P* = 0.29; Fig. 2), and approximately 20% in the association with externalizing problems among male offspring (PE = 23%, *P* = 0.26; Fig. 2), though the mediating effect was not statistically significant. In the association between adolescent motherhood and worse cognitive function, family environment only mediated approximately 10% of the association (Fig. 2).

In this study, adolescent mothers had similar or lower rates of gestational hypertension, gestational diabetes mellitus, preterm birth, and low birth weight than mothers aged 20–35 years. (Table 1). Thus, adverse pregnancy outcomes were unlikely to mediate the observed associations.

**Fig. 3:**
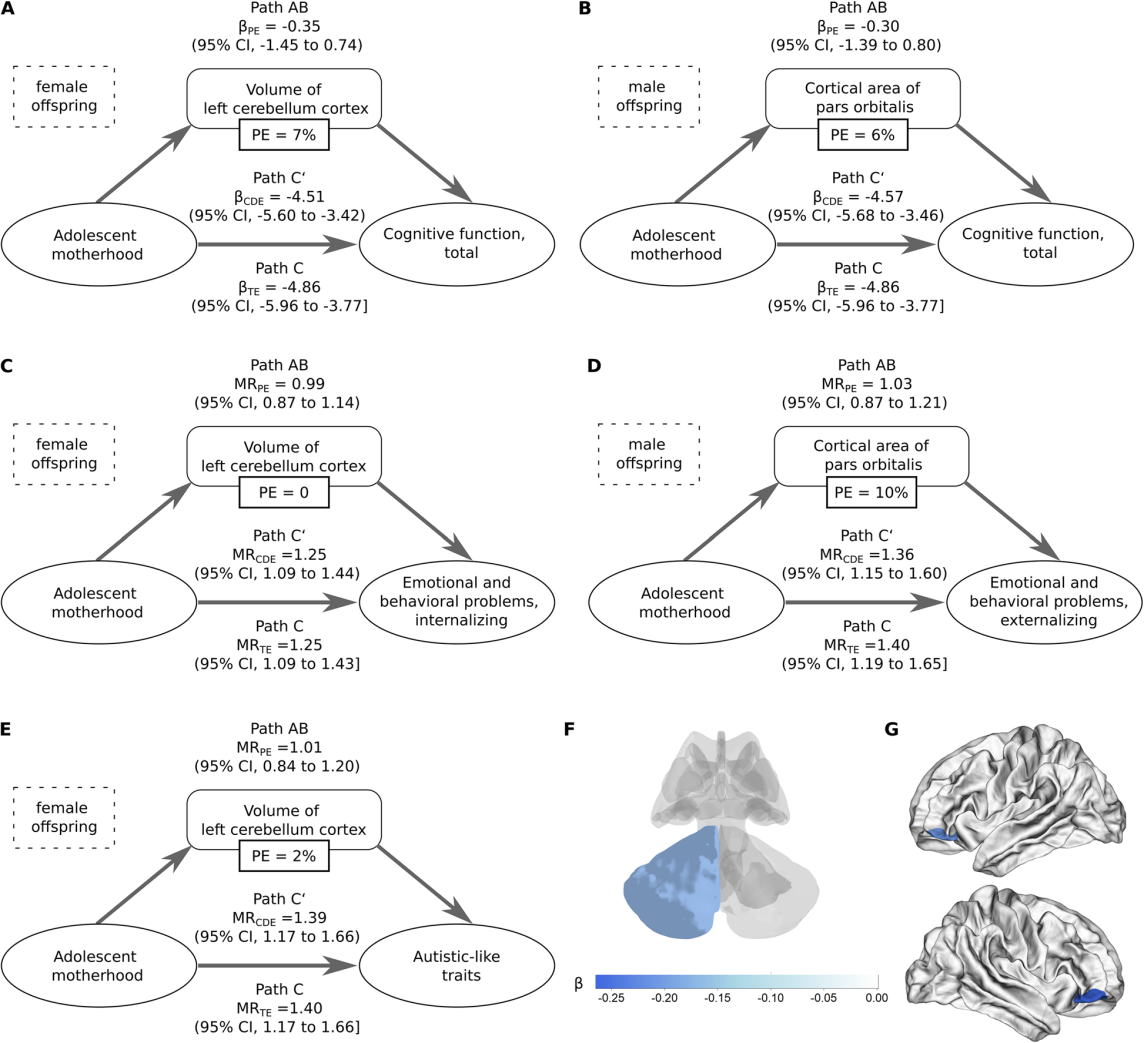
Mediation analysis of altered brain morphology in explaining the association of adolescent motherhood with **A** cognitive function in female offspring; **B** cognitive function in male offspring; **C** internalizing problems in female offspring; **D** externalizing problems in male offspring; and **E** autistic-like traits in female offspring. **F** Posterial view of the cerebellum cortex. **G** Lateral view of the pars orbitalis region. For NIH Toolbox in females (**A**), the indirect path (AB) shows that the volume of the left cerebellum cortex could mediate part of the effect of adolescent motherhood. Path C shows that the risk estimate (β) of adolescent motherhood on the NIH Toolbox was prominent when the volume of the left cerebellum cortex was not taken into account. Path C′ indicates the direct effect of adolescent motherhood on NIH Toolbox controlling for the mediator (the volume of the left cerebellum cortex). Path C’ shows some reduction in the risk estimate when the effect of the volume of the left cerebellum cortex was taken into account. Path AB indicates the extent to which taking the volume of the left cerebellum cortex into account can explain the 7% effect of the adolescent motherhood on NIH Toolbox, though insignificant because the confidence interval of β_PE_ crossed unity. Other mediation analyses were presented in a similar way. MR, mean ratio; TE, total effect; CDE, controlled direct effect; PE, proportion eliminated. Proportion eliminated = (MR_TE_ − MR_CDE_)/(MR_TE_ − 1), or (β_TE_ − β_CDE_)/β_TE_. Proportion eliminated is only presented if the βs of CDE and PE were in the same direction. Cognitive function was measured by the NIH Toolbox. Autistic-like traits were measured by the SSRS. Emotional and behavioral problems were measured by the CBCL

We investigated the sex-specific association between adolescent motherhood and brain morphometric measures while additionally adjusting for the family environment score (Additional file 1: Table S5–S14). Among male offspring, having a mother aged less than 20 years at birth was associated with smaller cortical area of right and left pars orbitalis (both FDR < 0.05; Cohen’s *D*, − 0.26 and − 0.21, respectively; Additional file 1: Table S5), which was associated with cognitive function and externalizing problems in male (Additional file 1: Table S13). Among female offspring, having a mother aged less than 20 years at birth was marginally associated with a smaller volume of left cerebellum cortex (FDR = 0.06; Cohen’s *D*, − 0.16; Additional file 1: Table S12), which was associated with cognitive function and autistic-like traits in females (Additional file 1: Table S13). Mediation analyses showed that these alterations in brain structures mediated less than 10% of the association between adolescent motherhood and neurodevelopmental outcomes (Fig. 3). We observed no association of adolescent motherhood with total cortical volume, total cortical area, altered cortical thickness in specific brain regions, or volume in specific subcortical regions (Additional file 1: Table S5–12). The risk estimates from the subgroup analysis by study sites were comparable to our main analysis in direction and magnitude, despite some non-significant associations (Additional file 1: Table S14). The study on 2-year follow-up MRI data also showed similar risk estimates (Additional file 1: Table S14).

## Discussion

This study presents compelling evidence of sex-specific associations between adolescent motherhood and long-term neurocognitive outcomes in offspring during late childhood. In both male and female offspring, poorer cognitive function was observed. Moreover, male offspring were more prone to externalizing problems, while female offspring showed increased internalizing issues and autistic-like traits. Significantly, stressful family environments mediated a substantial proportion of these outcomes, with more than 70% in the case of internalizing problems in female offspring, and 20–30% of the association with externalizing problems in male offspring and autistic-like traits in female offspring. Adolescent motherhood was associated with specific brain morphology changes even adjusted for the family environment score, although these did not serve as mediators in our study.

In the United States, adolescent birth rates have been in steady decline since the 1990s, reaching around 13.9 per 1000 live births in 2021 [32]. This trend suggests that contemporary adolescent mothers are facing different selection pressures and societal factors, such as reduced welfare support [33, 34], compared to their counterparts three decades ago. Furthermore, adolescent mothers in the USA generally have better pregnancy outcomes than those in low- and middle-income countries [32, 35, 36]. These contextual elements should be considered when interpreting our findings, particularly since our study did not observe a significant role for adverse birth outcomes like preterm births or low birth weights in affecting neurodevelopmental outcomes [35]. Additionally, our findings provide contemporary evidence in a typical industrialized country, which may help to inform policy-making decisions in similar settings.

In accordance with previous studies, this study found an association between adolescent motherhood and cognitive deficits in offspring [5, 37–39]. As expected, when parental income level was included as a covariate, this association was significantly attenuated, underscoring the mediating role of socioeconomic status [40]. Other potential underlying mechanisms, such as in utero exposure to stressors [41, 42], perinatal emotional difficulties [7], and breastfeeding [37], require further exploration. Also, we observed a limited mediating role of altered brain structure. Our results indicated that the reported mediating role of lower volumes and areas of specific cortical regions might be confounded by the family environment [9].

A unique aspect of our study is the evidence of sex-specific differences in emotional and behavioral outcomes [3, 4, 10, 43]. Notably, we found that male offspring predominantly exhibited externalizing problems, whereas female offspring were more prone to internalizing issues. This contrasts with a comprehensive meta-analysis involving over 130,000 children, which suggested a link between adolescent motherhood and externalizing problems in offspring, but did not identify sex as a moderating factor in its sub-analysis of approximately 70,000 children [10]. The discrepancy between these findings may be attributed to the heterogeneity inherent in different studies, as highlighted by the wide range of risk estimates across existing research [10]. Although previous studies have reported a general association between adolescent motherhood and internalizing problems in offspring [11, 44], they have largely neglected to examine potential sex differences. Our study posits that the divergent mediation effects observed—stressful family environments playing a significant role in females’ internalizing problems and males’ externalizing problems—may mirror the distinct coping strategies employed by males and females when faced with stress [45, 46]. Specifically, females may be more vulnerable to emotional and relational stressors, manifesting as internalizing problems, whereas males may exhibit outward behaviors under stress, leading to externalizing issues [47]. These insights underscore the potential efficacy of family-centered interventions in mitigating these problems among children of adolescent mothers.

Interestingly, our study reveals a sex-specific correlation between adolescent motherhood and the prevalence of autistic-like traits, with this pattern observed exclusively in female offspring. Approximately 30% of this association can be accounted for by the influence of a stressful family environment. This complements a population-based study conducted in the Netherlands, which found a relationship between younger maternal age and an increase in autistic-like traits in children at the age of 13, although that study did not undertake a sex-specific analysis [11]. These findings suggest the possibility of unique underlying mechanisms that predispose girls in late childhood to develop autistic-like traits when born to adolescent mothers. Nevertheless, it should be noted that the existing literature on the link between younger maternal age and autism spectrum disorders is far from uniform [48–50]. As such, future research involving a greater number of adolescent parents is essential to substantiate our observations.

Our research indicates that adolescent motherhood may exert specific influences on offspring neurodevelopment, as evidenced by changes in brain structures [9, 51]. Notably, various metrics associated with brain structures, such as reductions in the cortical areas of the orbitofrontal cortex and anterior cingulate cortex, have also been linked to stressful family environment [52]. Upon adjusting for familial stress factors, we found that adolescent motherhood remains correlated with certain brain morphometric indicators, i.e., a smaller cortical area of the pars orbitalis in male offspring and a reduced volume of the left cerebellum cortex in females. The pars orbitalis is known as a neural hub involved in integrating the semantic and expressive aspects of communication [53]. The decreased cortical area of the pars orbitalis, which plays a crucial role in language and comprehension, may be related to the elevated rates of externalizing problems observed in male offspring in the current study. Future research could investigate deeper into the social functioning of males born to adolescent mothers, with a focus on longer-term follow-up. Conversely, the role of the cerebellum cortex in executive, emotional, and social-related networks in children could be pertinent to the internalizing issues and autistic-like traits found in female offspring [54]. A previous study reported an association between white matter integrity in the cerebellum and internalizing problems in school-aged girls [55]. Abnormal cerebellar development has also been noted in autistic children [56]. Cerebellar injuries in children can lead to the Cerebellar Cognitive Affective Syndrome, characterized by impairments in executive function, personality change, language production deficits, and visual–spatial disorganization [57]. While our study does not establish a mediating role for these brain structure changes, it underscores the need for future longitudinal studies to explore their developmental implications.

Our study has several strengths. First, this study involved a large sample size of nearly 10,000 children with multiple behavioral measures and neuroimage data. This provided sufficient statistical power to investigate the association and permitted mediation analyses to reveal potential underlying mechanisms. Second, the study provides novel evidence of sex-specific associations in both neurodevelopmental outcomes and brain morphometrics, enriching our understanding of the multifaceted impact of adolescent motherhood.

However, there are limitations to consider. First, we were unable to disentangle the effect of adolescent fatherhood due to the majority (82%, 284 out of 347) of the adolescent fathers having partners also under the age of 20. Nevertheless, our analyses adjusted for this factor, and the results remain solid. Second, the socioeconomic covariates, such as parental income level and social disparity were closely related to adolescent motherhood, serving as both potential confounders and mediators. This complicates the task of isolating the unique risks attributable adolescent motherhood. In our analyses, we considered parental income level as a confounder, which might induce overadjustment. Third, our study lacked information on maternal characteristics prior to pregnancy such as body mass index, pre-gestational hypertension, and diabetes. These factors could affect the neurodevelopmental outcomes of children and introduce potential confounding effects in our estimated associations. Fourth, the limited sample size (*N* < 10) of autistic children born to adolescent mothers precluded further in-depth analysis of this specific clinical outcome. Fifth, in our mediation analysis, we explored the role of family environment in the relationship between adolescent motherhood and the presence of externalizing symptoms in males, as well as autistic-like traits in females. Our findings indicated potential mediation effects, with PEs ranging from 20 to 30%. However, these mediating effects did not reach statistical significance. This suggests that while the family environment may play a role in this association, the evidence is not robust enough to draw definitive conclusions. Consequently, further research employing a larger sample size is essential to substantiate these preliminary findings and fully understand the mediating role of family environment in these associations. Lastly, we lack data that could illuminate the direct mechanism linking adolescent motherhood to neurodevelopmental outcomes once family conflict and neuroanatomical changes are considered. Therefore, additional studies are needed to explore other plausible mediators, such as prenatal exposure to stressors or maternal emotional challenges [41, 42].

## Conclusions

In conclusion, our study offers evidence of the substantial influence of adolescent motherhood on the neurodevelopment of offspring. Using a large sample and comprehensive behavioral and neuroimaging data, we found that adolescent motherhood was associated with particular brain morphometric changes, externalizing problems in male offspring, and internalizing issues and autistic-like traits in females. These sex-specific findings unveil new dimensions of the impact of adolescent motherhood, thereby contributing to a more comprehensive understanding of the far-reaching consequences of adolescent motherhood. Our findings underscore the need for sex-tailored interventions and support the theory that the family environment plays a critical role in moderating the neurodevelopmental outcomes associated with adolescent motherhood. Our findings not only hold academic value but also have the potential to inform policy decisions, as they highlight a public health issue that requires attention and action across various socioeconomic contexts worldwide.

### Supplementary Information

Additional file 1: Fig. S1. Structural equation model of family environment. Fig. S2. Causal diagram showing selection of covariates for analyses. Fig. S3. Associations of adolescent motherhood with A) cognitive function, B) externalizing problems in male offspring, and C) internalizing problems in female offspring, and D) autistic-like traits in female offspring, treating maternal age as a continuous variable. Fig. S4. Associations of adolescent motherhood with seven tasks of NIH Toolbox cognition measures. Fig. S5. Associations of adolescent motherhood with subdomains of Child Behavioral Checklist (CBCL). Table S1. Coding of covariates used in this study. Table S2. E-values of the associations between adolescent motherhood and neurodevelopmental outcomes. Table S3. Associations between adolescent motherhood and neurodevelopmental outcomes, additionally adjusted for birth year. Table S4. Associations between family environment and neurodevelopmental outcomes. Table S5. Associations between adolescent motherhood and cortical areas in male offspring. Table S6. Associations between adolescent motherhood and cortical areas in female offspring. Table S7. Associations between adolescent motherhood and cortical volumes in male offspring. Table S8. Associations between adolescent motherhood and cortical volumes in female offspring. Table S9. Associations between adolescent motherhood and cortical thickness in male offspring. Table S10. Associations between adolescent motherhood and cortical thickness in female offspring. Table S11. Associations between adolescent motherhood and subcortical volumes in male offspring. Table S12. Associations between adolescent motherhood and subcortical volumes in female offspring. Table S13. Associations between brain morphology that were associated with adolescent motherhood and neurodevelopmental outcomes. Table S14. Associations between adolescent motherhood and brain morphology in two randomly selected subgroups at baseline and 2-year follow-up. Additional Methods S1. Model selection for outcomes.

## Data Availability

Data are publicly released on an annual basis through the National Institute of Mental Health (NIMH) data archive (NDA, https://nda.nih.gov/abcd). The ABCD study are openly available to qualified researchers for free. Access can be requested at https://nda.nih.gov/abcd/request-access. The data that support the findings of this study are openly available in the ABCD Dataset Data Release 5.0. An NDA study has been created for the data used in this report and code for the replication of analyses conducted in the manuscript can be retrieved under the doi: 10.15154/h1py-4685.

## Abbreviations

ABCD: Adolescent Brain Cognitive Development
CBCL: Child Behavioral Checklist
FDR: False discovery rate
MRI: Magnetic resonance imaging
PE: Proportion eliminated
SD: Standard deviation
SSRS: Short-Social Responsiveness Scale
