# Supplementary material for: Sex-specific associations of adolescent motherhood with cognitive function, behavioral problems, and autistic-like traits in offspring and the mediating roles of family conflict and altered brain structure
Source: BMC Med. 2024 Jun 5;22:226. doi: 10.1186/s12916-024-03442-8 (PMC11155128; doi:10.1186/s12916-024-03442-8)
Supplement: Supplementary file 1 — Additional file 1: Fig. S1. Structural equation model of family environment. Fig. S2. Causal diagram showing selection of covariates for analyses. Fig. S3. Associations of adolescent motherhood with A) cognitive function, B) externalizing problems in male offspring, and C) internalizing problems in female offspring, and D) autistic-like traits in female offspring, treating maternal age as a continuous variable. Fig. S4. Associations of adolescent motherhood with seven tasks of NIH Toolbox cognition measures. Fig. S5. Associations of adolescent motherhood with subdomains of Child Behavioral Checklist (CBCL). Table S1. Coding of covariates used in this study. Table S2. E-values of the associations between adolescent motherhood and neurodevelopmental outcomes. Table S3. Associations between adolescent motherhood and neurodevelopmental outcomes, additionally adjusted for birth year. Table S4. Associations between family environment and neurodevelopmental outcomes. Table S5. Associations between adolescent motherhood and cortical areas in male offspring. Table S6. Associations between adolescent motherhood and cortical areas in female offspring. Table S7. Associations between adolescent motherhood and cortical volumes in male offspring. Table S8. Associations between adolescent motherhood and cortical volumes in female offspring. Table S9. Associations between adolescent motherhood and cortical thickness in male offspring. Table S10. Associations between adolescent motherhood and cortical thickness in female offspring. Table S11. Associations between adolescent motherhood and subcortical volumes in male offspring. Table S12. Associations between adolescent motherhood and subcortical volumes in female offspring. Table S13. Associations between brain morphology that were associated with adolescent motherhood and neurodevelopmental outcomes. Table S14. Associations between adolescent motherhood and brain morphology in two randomly selected subgroups at baseline and 2-year f [file 12916_2024_3442_MOESM1_ESM.docx]

**Additional File 1: Figures S1-S5, Tables S1-S14, and Additional Methods S1.**

Supplementary information for the paper

*“Sex-specific associations of* *adolescent motherhood with cognitive function, behavioral problems, and autistic-like traits in offspring and the mediating roles of family conflict and altered brain structure”*

Authors: Tai Ren, Lingli Zhang, Yongjie Liu, Qingli Zhang, Yunjun Sun, Wei Zhou, Like Huang, Ming Wang, Yiwei Pu, Runqi Huang, Jingyu Chen, Hua He, Tailin Zhu, Susu Wang, Weiran Chen, Qianlong Zhang, Wenchong Du, Qiang Luo, Fei Li

Correspondence: Dr. Fei Li, [feili@shsmu.edu.cn](mailto:feili@shsmu.edu.cn), Ministry of Education - Shanghai Key Laboratory of Children’s Environmental Health & Department of Developmental and Behavioural Paediatric & Child Primary Care, Xinhua Hospital Affiliated to Shanghai Jiao Tong University School of Medicine, China; Dr. Qiang Luo, [qluo@fudan.edu.cn](mailto:qluo@fudan.edu.cn), Institute of Science and Technology for Brain-Inspired Intelligence, Ministry of Education-Key Laboratory of Computational Neuroscience and Brain-Inspired Intelligence, Fudan University, China; Dr. Wenchong Du, [vivienne.du@ntu.ac.uk](mailto:vivienne.du@ntu.ac.uk), NTU Psychology, School of Social Sciences, Nottingham Trent University, UK.

**Table of Contents**

[Figure S1. Structural equation model of family environment. 3](#_Toc165897748)

[Figure S2. Causal diagram showing selection of covariates for analyses. 4](#_Toc165897749)

[Figure S3. Associations of adolescent motherhood with A) cognitive function, B) externalizing problems in male offspring, and C) internalizing problems in female offspring, and D) autistic-like traits in female offspring, treating maternal age as a continuous variable. 5](#_Toc165897750)

[Figure S4. Associations of adolescent motherhood with seven tasks of NIH Toolbox cognition measures. 6](#_Toc165897751)

[Figure S5. Associations of adolescent motherhood with subdomains of Child Behavioral Checklist (CBCL). 7](#_Toc165897752)

[Table S1. Coding of covariates used in this study 8](#_Toc165897753)

[Table S2. E-values of the associations between adolescent motherhood and neurodevelopmental outcomes. 9](#_Toc165897754)

[Table S3. Associations between adolescent motherhood and neurodevelopmental outcomes, additionally adjusted for birth year. 10](#_Toc165897755)

[Table S4. Associations between family environment and neurodevelopmental outcomes. 11](#_Toc165897756)

[Table S5. Associations between adolescent motherhood and cortical areas in male offspring. 12](#_Toc165897757)

[Table S6. Associations between adolescent motherhood and cortical areas in female offspring. 14](#_Toc165897758)

[Table S7. Associations between adolescent motherhood and cortical volumes in male offspring. 16](#_Toc165897759)

[Table S8. Associations between adolescent motherhood and cortical volumes in female offspring. 18](#_Toc165897760)

[Table S9. Associations between adolescent motherhood and cortical thickness in male offspring. 20](#_Toc165897761)

[Table S10. Associations between adolescent motherhood and cortical thickness in female offspring. 22](#_Toc165897762)

[Table S11. Associations between adolescent motherhood and subcortical volumes in male offspring. 24](#_Toc165897763)

[Table S12. Associations between adolescent motherhood and subcortical volumes in female offspring. 25](#_Toc165897764)

[Table S13. Associations between brain morphology that were associated with adolescent motherhood and neurodevelopmental outcomes. 27](#_Toc165897765)

[Table S14. Associations between adolescent motherhood and brain morphology in two randomly selected subgroups at baseline and 2-year follow-up. 28](#_Toc165897766)

[Additional Methods S1. Model selection for outcomes. 29](#_Toc165897767)


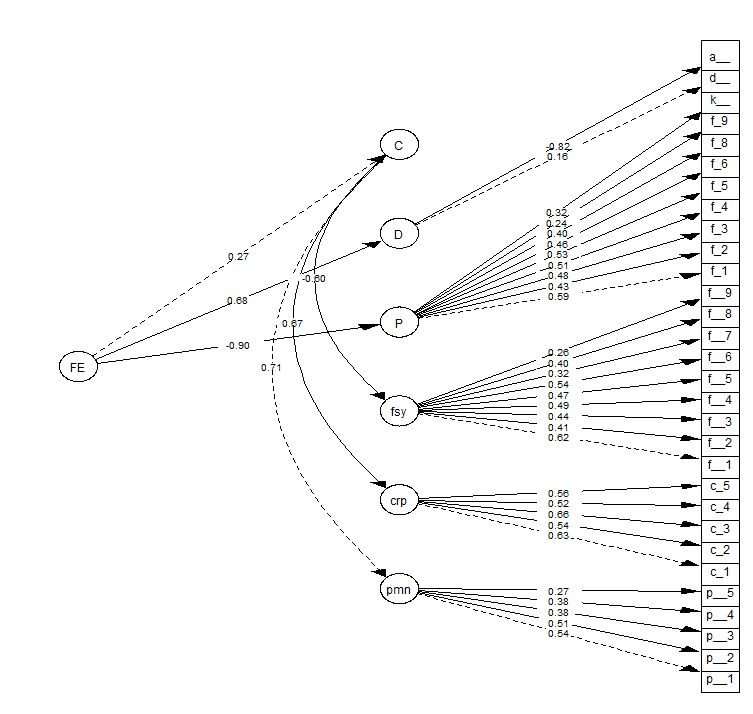


# Figure S1. Structural equation model of family environment.

FE, Family Environment; C, Child-report latent variable; D, Demographic and parental latent variable; P, Parent-reported latent variable; fsy, Family Environment Scale youth report latent variable; crp, Child report of parent behavior inventory latent variable; pmn, Parental monitoring latent variable; a__, parental psychopathology; d__, parental separation; k__ (under ’P’ category), KSADS item on parent-child conflict; f_1 to f_9 (excluding f_8; under ’P’ category), Items of Conflict scale of FES parent report; f__1 to f__9 (under ’fsy’ category), items of Conflict Scale of Family Environment Scale youth report; c_1 to c_5, Items of Parental acceptance scale of Child report of parent behavior inventory; p__1 to p__5, items of Parental Monitory Scale.


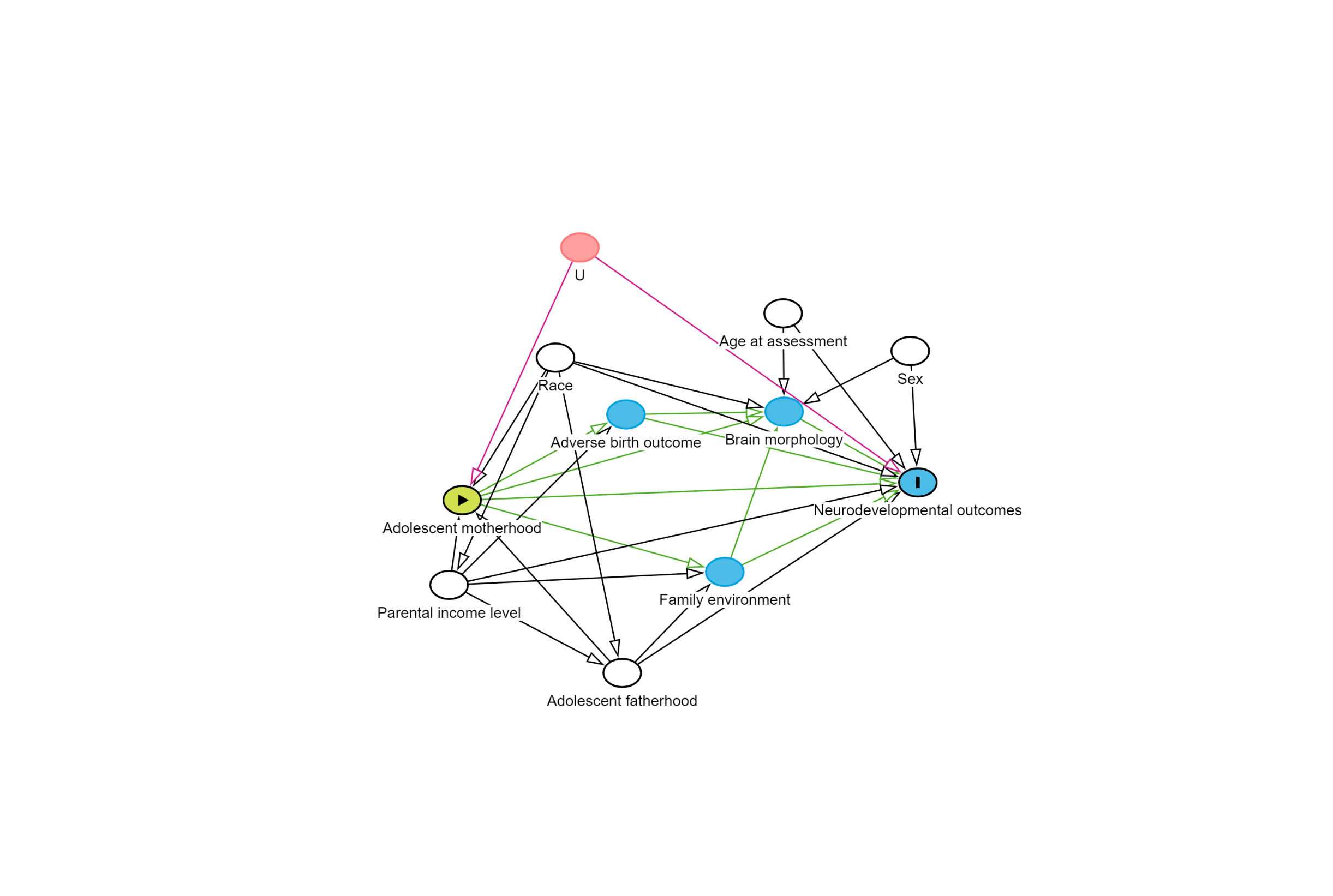


# Figure S2. Causal diagram showing selection of covariates for analyses.

U, unmeasured confounders.

In this study, we considered parental income level, adolescent fatherhood, and race as confounders in the association between adolescent motherhood and offspring’s neurodevelopmental outcomes. Sex and age at assessment were also adjusted. We considered adverse birth outcome, brain morphology change, and family environment as mediators.


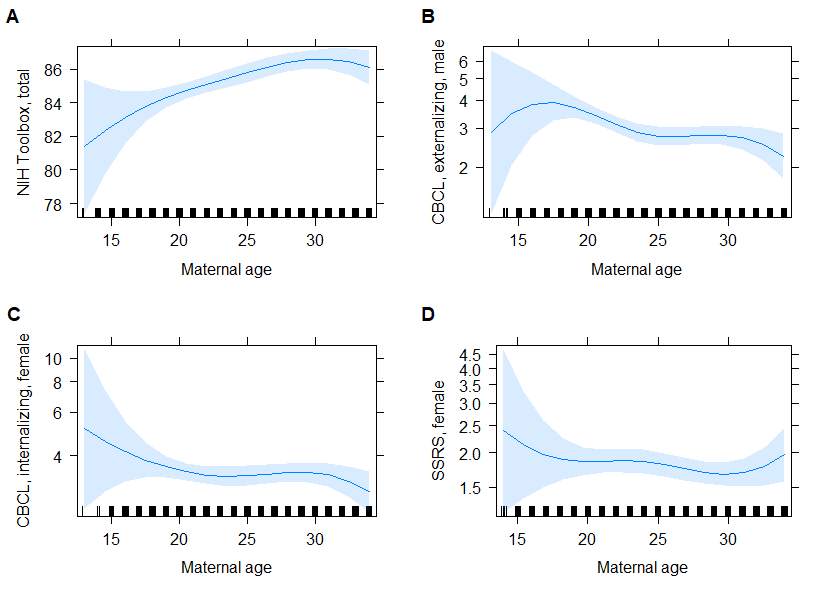


# Figure S3. Associations of adolescent motherhood with A) cognitive function, B) externalizing problems in male offspring, and C) internalizing problems in female offspring, and D) autistic-like traits in female offspring, treating maternal age as a continuous variable.

All models were fitted adjusted for paternal age less than 20 years at birth, race, sex, age at outcome assessment, and parental income level.


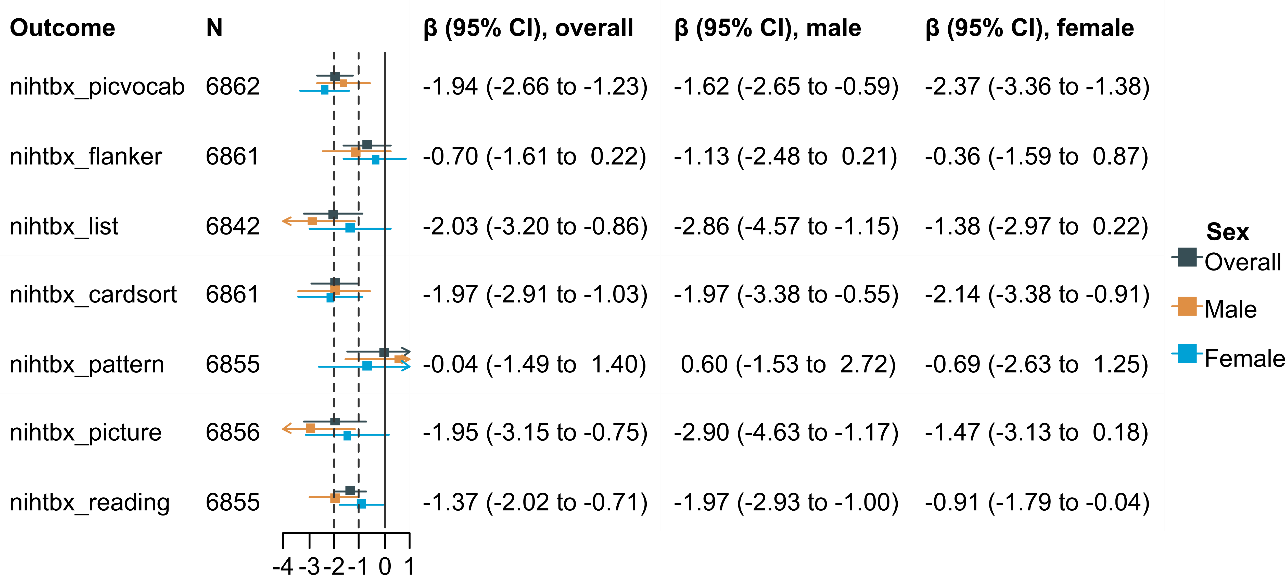


# Figure S4. Associations of adolescent motherhood with seven tasks of NIH Toolbox cognition measures.

All models were fitted adjusted for paternal age less than 20 years at birth, race, sex, age at outcome assessment, and parental income level.


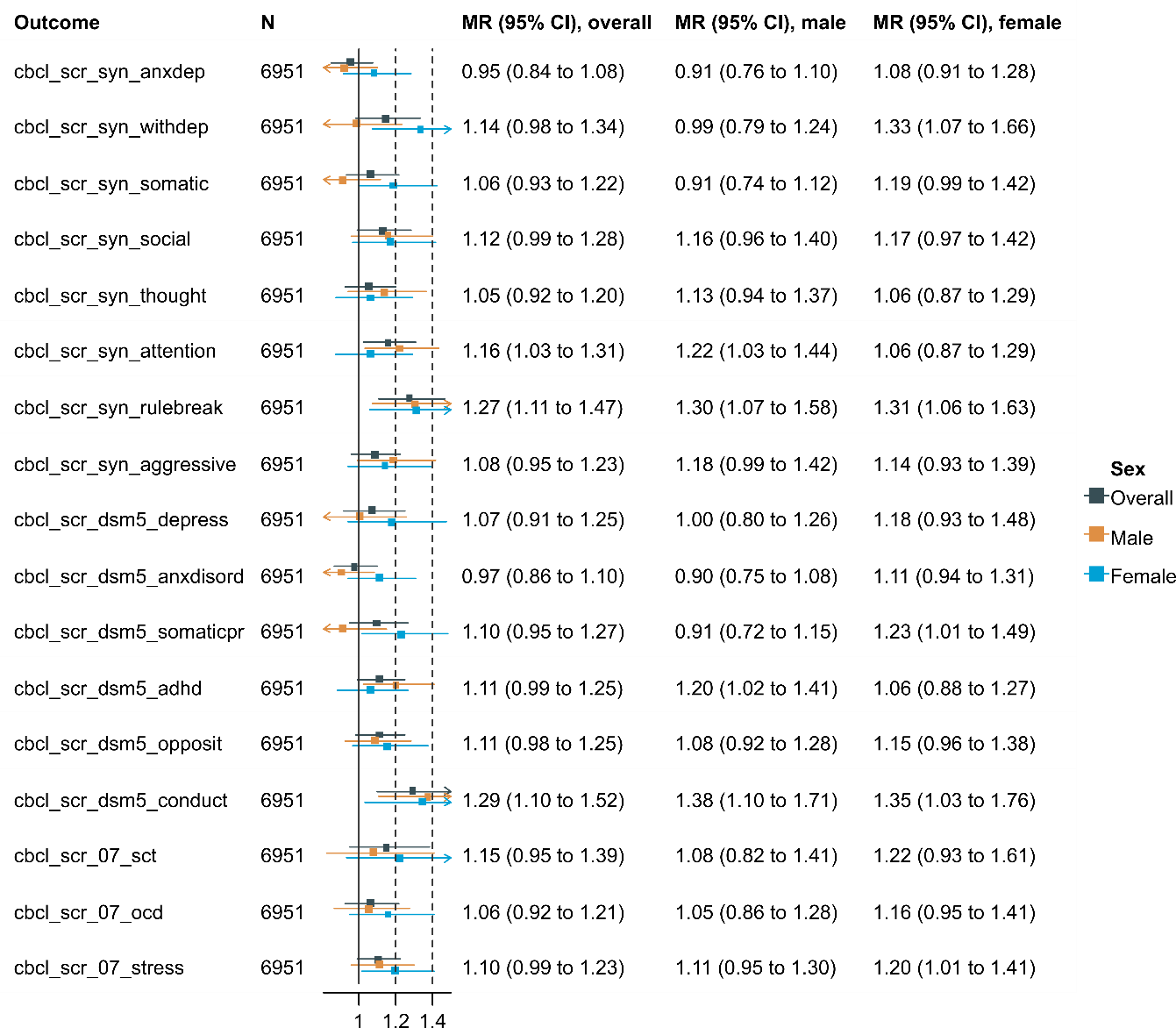


# Figure S5. Associations of adolescent motherhood with subdomains of Child Behavioral Checklist (CBCL).

All models were fitted adjusted for paternal age less than 20 years at birth, race, sex, age at outcome assessment, and parental income level.

# Table S1. Coding of covariates used in this study

| **Variable, label** | **Coding** |
| --- | --- |
| Family income |  |
| 1 | Less than $5,000 |
| 2 | $5,000 through $11,999 |
| 3 | $12,000 through $15,999 |
| 4 | $16,000 through $24,999 |
| 5 | $25,000 through $34,999 |
| 6 | $35,000 through $49,999 |
| 7 | $50,000 through $74,999 |
| 8 | $75,000 through $99,999 |
| 9 | $100,000 through $199,999 |
| 10 | $200,000 and greater |

# Table S2. E-values of the associations between adolescent motherhood and neurodevelopmental outcomes.

| **Outcome** | **Sex** | **E-value** | **E-value, lower confidence** |
| --- | --- | --- | --- |
| NIH Toolbox | Male | 1.90 | 1.57 |
| NIH Toolbox | Female | 1.74 | 1.41 |
| CBCL externalizing | Male | 1.88 | 1.37 |
| CBCL internalizing | Female | 1.54 | 1.00 |
| SSRS | Female | 1.74 | 1.11 |

SSRS, Short-Social Responsiveness Scale; CBCL, Child Behavioral Checklist.

# Table S3. Associations between adolescent motherhood and neurodevelopmental outcomes, additionally adjusted for birth year.

| **Outcome** | **Sex** | **N** | **Adjusted β, 95% CI^*^** |
| --- | --- | --- | --- |
| NIH Toolbox | Male | 3555 | -2.60 (-3.76 to -1.43) |
| NIH Toolbox | Female | 3265 | -1.90 (-2.98 to -0.83) |
| **Outcome** | **Sex** | **N** | **Adjusted mean ratio, 95% CI^*^** |
| CBCL externalizing | Male | 3617 | 1.27 (1.07 to 1.51) |
| CBCL externalizing | Female | 3334 | 1.17 (0.97 to 1.42) |
| CBCL internalizing | Male | 3617 | 0.92 (0.79 to 1.07) |
| CBCL internalizing | Female | 3334 | 1.15 (0.99 to 1.33) |
| SSRS | Male | 3138 | 0.98 (0.81 to 1.18) |
| SSRS | Female | 2866 | 1.22 (1.01 to 1.48) |

SSRS, Short-Social Responsiveness Scale; CBCL, Child Behavioral Checklist.

**^*^** Adjusted for paternal age less than 20 years at birth, race, sex, age at outcome assessment, parental income level, and birth year.

# Table S4. Associations between family environment and neurodevelopmental outcomes.

| **Outcome** | **Sex** | **N** | **Adjusted β, 95% CI^*^** |
| --- | --- | --- | --- |
| NIH Toolbox | Male | 3555 | 0.76 (0.23 to 1.29) |
| NIH Toolbox | Female | 3265 | 0.98 (0.44 to 1.51) |
| **Outcome** | **Sex** | **N** | **Adjusted mean ratio, 95% CI^*^** |
| CBCL externalizing | Male | 3617 | 0.28 (0.26 to 0.30) |
| CBCL externalizing | Female | 3334 | 0.26 (0.24 to 0.28) |
| CBCL internalizing | Male | 3617 | 0.37 (0.34 to 0.39) |
| CBCL internalizing | Female | 3334 | 0.38 (0.35 to 0.40) |
| SSRS | Male | 3138 | 0.51 (0.47 to 0.55) |
| SSRS | Female | 2866 | 0.47 (0.43 to 0.51) |

SSRS, Short-Social Responsiveness Scale; CBCL, Child Behavioral Checklist.

**^*^** Adjusted for paternal age less than 20 years at birth, race, sex, age at outcome assessment, and parental income level.

# Table S5. Associations between adolescent motherhood and cortical areas in male offspring.

| **Outcome** | **N** | **β** | **t** | **P** | **FDR** |
| --- | --- | --- | --- | --- | --- |
| Cortical area lh-Banks of Superior Temporal Sulcus | 3267 | -0.016 | -0.221 | 0.825 | 0.920 |
| Cortical area lh-caudalanteriorcingulate | 3267 | -0.146 | -1.928 | 0.055 | 0.351 |
| Cortical area lh-caudalmiddlefrontal | 3267 | -0.071 | -1.044 | 0.297 | 0.777 |
| Cortical area lh-cuneus | 3267 | -0.061 | -0.837 | 0.403 | 0.808 |
| Cortical area lh-entorhinal | 3267 | -0.154 | -1.912 | 0.057 | 0.351 |
| Cortical area lh-fusiform | 3267 | -0.037 | -0.640 | 0.522 | 0.808 |
| Cortical area lh-inferiorparietal | 3267 | 0.032 | 0.477 | 0.634 | 0.808 |
| Cortical area lh-inferiortemporal | 3267 | 0.035 | 0.599 | 0.549 | 0.808 |
| Cortical area lh-isthmuscingulate | 3267 | -0.058 | -0.834 | 0.405 | 0.808 |
| Cortical area lh-lateraloccipital | 3267 | -0.115 | -1.755 | 0.080 | 0.454 |
| Cortical area lh-lateralorbitofrontal | 3267 | -0.146 | -2.600 | 0.010 | 0.133 |
| Cortical area lh-lingual | 3267 | -0.193 | -2.637 | 0.009 | 0.133 |
| Cortical area lh-medialorbitofrontal | 3267 | -0.046 | -0.754 | 0.452 | 0.808 |
| Cortical area lh-middletemporal | 3267 | -0.009 | -0.148 | 0.883 | 0.927 |
| Cortical area lh-parahippocampal | 3267 | -0.033 | -0.466 | 0.642 | 0.808 |
| Cortical area lh-paracentral | 3267 | 0.053 | 0.741 | 0.459 | 0.808 |
| Cortical area lh-parsopercularis | 3267 | -0.079 | -1.072 | 0.285 | 0.777 |
| **Cortical area lh-parsorbitalis** | **3267** | **-0.206** | **-3.249** | **0.001** | **0.043** |
| Cortical area lh-parstriangularis | 3267 | -0.062 | -0.851 | 0.396 | 0.808 |
| Cortical area lh-pericalcarine | 3267 | -0.105 | -1.382 | 0.168 | 0.549 |
| Cortical area lh-postcentral | 3267 | -0.043 | -0.710 | 0.478 | 0.808 |
| Cortical area lh-posteriorcingulate | 3267 | -0.038 | -0.543 | 0.588 | 0.808 |
| Cortical area lh-precentral | 3267 | -0.010 | -0.161 | 0.872 | 0.927 |
| Cortical area lh-precuneus | 3267 | 0.104 | 1.692 | 0.092 | 0.479 |
| Cortical area lh-rostralanteriorcingulate | 3267 | -0.167 | -2.626 | 0.009 | 0.133 |
| Cortical area lh-rostralmiddlefrontal | 3267 | -0.085 | -1.403 | 0.162 | 0.549 |
| Cortical area lh-superiorfrontal | 3267 | -0.057 | -1.003 | 0.317 | 0.797 |
| Cortical area lh-superiorparietal | 3267 | 0.018 | 0.286 | 0.775 | 0.893 |
| Cortical area lh-superiortemporal | 3267 | -0.044 | -0.732 | 0.465 | 0.808 |
| Cortical area lh-supramarginal | 3267 | 0.043 | 0.638 | 0.524 | 0.808 |
| Cortical area lh-frontalpole | 3267 | -0.150 | -2.247 | 0.025 | 0.238 |
| Cortical area lh-temporalpole | 3267 | -0.105 | -1.501 | 0.134 | 0.539 |
| Cortical area lh-transversetemporal | 3267 | -0.048 | -0.621 | 0.535 | 0.808 |
| Cortical area lh-insula | 3267 | -0.001 | -0.009 | 0.993 | 0.993 |
| Cortical area rh-Banks of Superior Temporal Sulcus | 3267 | 0.051 | 0.714 | 0.476 | 0.808 |
| Cortical area rh-caudalanteriorcingulate | 3267 | 0.059 | 0.797 | 0.426 | 0.808 |
| Cortical area rh-caudalmiddlefrontal | 3267 | -0.034 | -0.501 | 0.617 | 0.808 |
| Cortical area rh-cuneus | 3267 | -0.079 | -1.092 | 0.276 | 0.777 |
| Cortical area rh-entorhinal | 3267 | -0.042 | -0.531 | 0.596 | 0.808 |
| Cortical area rh-fusiform | 3267 | -0.129 | -2.208 | 0.028 | 0.238 |
| Cortical area rh-inferiorparietal | 3267 | 0.009 | 0.144 | 0.886 | 0.927 |
| Cortical area rh-inferiortemporal | 3267 | -0.021 | -0.355 | 0.723 | 0.873 |
| Cortical area rh-isthmuscingulate | 3267 | -0.034 | -0.482 | 0.630 | 0.808 |
| Cortical area rh-lateraloccipital | 3267 | -0.007 | -0.102 | 0.919 | 0.947 |
| Cortical area rh-lateralorbitofrontal | 3267 | -0.143 | -2.476 | 0.014 | 0.156 |
| Cortical area rh-lingual | 3267 | -0.110 | -1.499 | 0.135 | 0.539 |
| Cortical area rh-medialorbitofrontal | 3267 | -0.089 | -1.564 | 0.119 | 0.539 |
| Cortical area rh-middletemporal | 3267 | 0.011 | 0.194 | 0.847 | 0.927 |
| Cortical area rh-parahippocampal | 3267 | -0.034 | -0.480 | 0.631 | 0.808 |
| Cortical area rh-paracentral | 3267 | 0.036 | 0.503 | 0.615 | 0.808 |
| Cortical area rh-parsopercularis | 3267 | -0.070 | -0.960 | 0.338 | 0.808 |
| **Cortical area rh-parsorbitalis** | **3267** | **-0.264** | **-4.035** | **<0.001** | **0.005** |
| Cortical area rh-parstriangularis | 3267 | -0.059 | -0.786 | 0.432 | 0.808 |
| Cortical area rh-pericalcarine | 3267 | -0.089 | -1.166 | 0.244 | 0.756 |
| Cortical area rh-postcentral | 3267 | -0.102 | -1.655 | 0.099 | 0.480 |
| Cortical area rh-posteriorcingulate | 3267 | -0.016 | -0.232 | 0.816 | 0.920 |
| Cortical area rh-precentral | 3267 | 0.021 | 0.343 | 0.732 | 0.873 |
| Cortical area rh-precuneus | 3267 | 0.034 | 0.571 | 0.569 | 0.808 |
| Cortical area rh-rostralanteriorcingulate | 3267 | -0.020 | -0.291 | 0.772 | 0.893 |
| Cortical area rh-rostralmiddlefrontal | 3267 | -0.085 | -1.377 | 0.169 | 0.549 |
| Cortical area rh-superiorfrontal | 3267 | -0.062 | -1.060 | 0.290 | 0.777 |
| Cortical area rh-superiorparietal | 3267 | 0.032 | 0.507 | 0.613 | 0.808 |
| Cortical area rh-superiortemporal | 3267 | 0.024 | 0.417 | 0.677 | 0.837 |
| Cortical area rh-supramarginal | 3267 | -0.063 | -0.944 | 0.346 | 0.808 |
| Cortical area rh-frontalpole | 3267 | -0.095 | -1.412 | 0.159 | 0.549 |
| Cortical area rh-temporalpole | 3267 | -0.153 | -2.104 | 0.036 | 0.273 |
| Cortical area rh-transversetemporal | 3267 | -0.005 | -0.067 | 0.947 | 0.961 |
| Cortical area rh-insula | 3267 | 0.047 | 0.747 | 0.456 | 0.808 |

All models were fitted adjusted for paternal age less than 20 years at birth, race, sex, age at outcome assessment, parental income level, family environment score, T1 image signal-to-noise, and intracranial volume.

# Table S6. Associations between adolescent motherhood and cortical areas in female offspring.

| **Outcome** | **N** | **β** | **t** | **P** | **FDR** |
| --- | --- | --- | --- | --- | --- |
| Cortical area lh-Banks of Superior Temporal Sulcus | 3037 | -0.084 | -1.340 | 0.181 | 0.536 |
| Cortical area lh-caudalanteriorcingulate | 3037 | -0.119 | -1.840 | 0.067 | 0.453 |
| Cortical area lh-caudalmiddlefrontal | 3037 | -0.035 | -0.568 | 0.571 | 0.731 |
| Cortical area lh-cuneus | 3037 | 0.030 | 0.441 | 0.659 | 0.792 |
| Cortical area lh-entorhinal | 3037 | -0.027 | -0.413 | 0.680 | 0.797 |
| Cortical area lh-fusiform | 3037 | 0.012 | 0.228 | 0.820 | 0.868 |
| Cortical area lh-inferiorparietal | 3037 | -0.038 | -0.621 | 0.535 | 0.731 |
| Cortical area lh-inferiortemporal | 3037 | 0.024 | 0.435 | 0.664 | 0.792 |
| Cortical area lh-isthmuscingulate | 3037 | -0.013 | -0.232 | 0.817 | 0.868 |
| Cortical area lh-lateraloccipital | 3037 | 0.006 | 0.099 | 0.921 | 0.921 |
| Cortical area lh-lateralorbitofrontal | 3037 | -0.072 | -1.396 | 0.164 | 0.536 |
| Cortical area lh-lingual | 3037 | 0.024 | 0.373 | 0.710 | 0.818 |
| Cortical area lh-medialorbitofrontal | 3037 | -0.087 | -1.701 | 0.090 | 0.508 |
| Cortical area lh-middletemporal | 3037 | 0.030 | 0.554 | 0.580 | 0.731 |
| Cortical area lh-parahippocampal | 3037 | 0.091 | 1.484 | 0.139 | 0.536 |
| Cortical area lh-paracentral | 3037 | -0.087 | -1.462 | 0.145 | 0.536 |
| Cortical area lh-parsopercularis | 3037 | -0.081 | -1.287 | 0.199 | 0.564 |
| Cortical area lh-parsorbitalis | 3037 | -0.065 | -1.106 | 0.269 | 0.572 |
| Cortical area lh-parstriangularis | 3037 | -0.075 | -1.181 | 0.239 | 0.572 |
| Cortical area lh-pericalcarine | 3037 | 0.050 | 0.698 | 0.486 | 0.718 |
| Cortical area lh-postcentral | 3037 | -0.137 | -2.617 | 0.009 | 0.210 |
| Cortical area lh-posteriorcingulate | 3037 | -0.047 | -0.804 | 0.422 | 0.696 |
| Cortical area lh-precentral | 3037 | -0.064 | -1.194 | 0.234 | 0.572 |
| Cortical area lh-precuneus | 3037 | -0.099 | -1.876 | 0.062 | 0.453 |
| Cortical area lh-rostralanteriorcingulate | 3037 | -0.191 | -3.226 | 0.001 | 0.094 |
| Cortical area lh-rostralmiddlefrontal | 3037 | 0.043 | 0.766 | 0.444 | 0.696 |
| Cortical area lh-superiorfrontal | 3037 | -0.040 | -0.786 | 0.432 | 0.696 |
| Cortical area lh-superiorparietal | 3037 | -0.158 | -2.662 | 0.008 | 0.210 |
| Cortical area lh-superiortemporal | 3037 | -0.031 | -0.594 | 0.553 | 0.731 |
| Cortical area lh-supramarginal | 3037 | -0.059 | -1.018 | 0.309 | 0.601 |
| Cortical area lh-frontalpole | 3037 | -0.081 | -1.340 | 0.181 | 0.536 |
| Cortical area lh-temporalpole | 3037 | 0.044 | 0.733 | 0.464 | 0.701 |
| Cortical area lh-transversetemporal | 3037 | -0.072 | -1.171 | 0.243 | 0.572 |
| Cortical area lh-insula | 3037 | -0.093 | -1.663 | 0.097 | 0.508 |
| Cortical area rh-Banks of Superior Temporal Sulcus | 3037 | -0.035 | -0.577 | 0.564 | 0.731 |
| Cortical area rh-caudalanteriorcingulate | 3037 | -0.012 | -0.175 | 0.861 | 0.887 |
| Cortical area rh-caudalmiddlefrontal | 3037 | -0.118 | -1.914 | 0.056 | 0.453 |
| Cortical area rh-cuneus | 3037 | -0.049 | -0.756 | 0.450 | 0.696 |
| Cortical area rh-entorhinal | 3037 | -0.099 | -1.546 | 0.123 | 0.523 |
| Cortical area rh-fusiform | 3037 | -0.107 | -2.069 | 0.039 | 0.382 |
| Cortical area rh-inferiorparietal | 3037 | -0.081 | -1.423 | 0.156 | 0.536 |
| Cortical area rh-inferiortemporal | 3037 | -0.025 | -0.452 | 0.652 | 0.792 |
| Cortical area rh-isthmuscingulate | 3037 | -0.066 | -1.114 | 0.266 | 0.572 |
| Cortical area rh-lateraloccipital | 3037 | -0.012 | -0.218 | 0.828 | 0.868 |
| Cortical area rh-lateralorbitofrontal | 3037 | -0.082 | -1.614 | 0.107 | 0.522 |
| Cortical area rh-lingual | 3037 | 0.079 | 1.213 | 0.226 | 0.572 |
| Cortical area rh-medialorbitofrontal | 3037 | -0.116 | -2.237 | 0.026 | 0.353 |
| Cortical area rh-middletemporal | 3037 | 0.058 | 1.147 | 0.252 | 0.572 |
| Cortical area rh-parahippocampal | 3037 | 0.062 | 1.022 | 0.307 | 0.601 |
| Cortical area rh-paracentral | 3037 | -0.050 | -0.836 | 0.404 | 0.696 |
| Cortical area rh-parsopercularis | 3037 | -0.083 | -1.352 | 0.177 | 0.536 |
| Cortical area rh-parsorbitalis | 3037 | -0.040 | -0.666 | 0.506 | 0.724 |
| Cortical area rh-parstriangularis | 3037 | 0.061 | 0.952 | 0.342 | 0.628 |
| Cortical area rh-pericalcarine | 3037 | 0.043 | 0.602 | 0.548 | 0.731 |
| Cortical area rh-postcentral | 3037 | -0.052 | -0.985 | 0.326 | 0.615 |
| Cortical area rh-posteriorcingulate | 3037 | -0.134 | -2.282 | 0.023 | 0.353 |
| Cortical area rh-precentral | 3037 | -0.012 | -0.216 | 0.829 | 0.868 |
| Cortical area rh-precuneus | 3037 | -0.110 | -2.096 | 0.037 | 0.382 |
| Cortical area rh-rostralanteriorcingulate | 3037 | -0.049 | -0.782 | 0.435 | 0.696 |
| Cortical area rh-rostralmiddlefrontal | 3037 | 0.038 | 0.658 | 0.511 | 0.724 |
| Cortical area rh-superiorfrontal | 3037 | -0.042 | -0.821 | 0.412 | 0.696 |
| Cortical area rh-superiorparietal | 3037 | -0.066 | -1.130 | 0.259 | 0.572 |
| Cortical area rh-superiortemporal | 3037 | 0.017 | 0.325 | 0.745 | 0.831 |
| Cortical area rh-supramarginal | 3037 | -0.099 | -1.725 | 0.085 | 0.508 |
| Cortical area rh-frontalpole | 3037 | -0.065 | -1.049 | 0.295 | 0.601 |
| Cortical area rh-temporalpole | 3037 | -0.096 | -1.575 | 0.116 | 0.523 |
| Cortical area rh-transversetemporal | 3037 | 0.020 | 0.335 | 0.738 | 0.831 |
| Cortical area rh-insula | 3037 | -0.008 | -0.138 | 0.890 | 0.904 |

All models were fitted adjusted for paternal age less than 20 years at birth, race, sex, age at outcome assessment, parental income level, family environment score, T1 image signal-to-noise, and intracranial volume.

# Table S7. Associations between adolescent motherhood and cortical volumes in male offspring.

| Outcome | N | β | t | P | FDR |
| --- | --- | --- | --- | --- | --- |
| Cortical volume lh-Banks of Superior Temporal Sulcus | 3267 | 0.004 | 0.054 | 0.957 | 0.981 |
| Cortical volume lh-caudalanteriorcingulate | 3267 | -0.106 | -1.386 | 0.167 | 0.679 |
| Cortical volume lh-caudalmiddlefrontal | 3267 | -0.025 | -0.351 | 0.726 | 0.943 |
| Cortical volume lh-cuneus | 3267 | -0.056 | -0.758 | 0.449 | 0.837 |
| Cortical volume lh-entorhinal | 3267 | -0.167 | -2.095 | 0.037 | 0.389 |
| Cortical volume lh-fusiform | 3267 | 0.014 | 0.230 | 0.818 | 0.951 |
| Cortical volume lh-inferiorparietal | 3267 | 0.063 | 0.918 | 0.359 | 0.837 |
| Cortical volume lh-inferiortemporal | 3267 | 0.048 | 0.771 | 0.441 | 0.837 |
| Cortical volume lh-isthmuscingulate | 3267 | -0.050 | -0.707 | 0.480 | 0.837 |
| Cortical volume lh-lateraloccipital | 3267 | -0.090 | -1.391 | 0.165 | 0.679 |
| Cortical volume lh-lateralorbitofrontal | 3267 | -0.129 | -2.275 | 0.024 | 0.389 |
| Cortical volume lh-lingual | 3267 | -0.127 | -1.775 | 0.077 | 0.515 |
| Cortical volume lh-medialorbitofrontal | 3267 | -0.054 | -0.888 | 0.375 | 0.837 |
| Cortical volume lh-middletemporal | 3267 | 0.016 | 0.257 | 0.798 | 0.951 |
| Cortical volume lh-parahippocampal | 3267 | -0.047 | -0.619 | 0.537 | 0.849 |
| Cortical volume lh-paracentral | 3267 | 0.121 | 1.696 | 0.091 | 0.515 |
| Cortical volume lh-parsopercularis | 3267 | -0.058 | -0.780 | 0.436 | 0.837 |
| Cortical volume lh-parsorbitalis | 3267 | -0.162 | -2.375 | 0.018 | 0.389 |
| Cortical volume lh-parstriangularis | 3267 | -0.006 | -0.074 | 0.941 | 0.981 |
| Cortical volume lh-pericalcarine | 3267 | -0.058 | -0.759 | 0.448 | 0.837 |
| Cortical volume lh-postcentral | 3267 | -0.026 | -0.406 | 0.685 | 0.943 |
| Cortical volume lh-posteriorcingulate | 3267 | 0.012 | 0.181 | 0.856 | 0.951 |
| Cortical volume lh-precentral | 3267 | 0.050 | 0.814 | 0.416 | 0.837 |
| Cortical volume lh-precuneus | 3267 | 0.133 | 2.174 | 0.030 | 0.389 |
| Cortical volume lh-rostralanteriorcingulate | 3267 | -0.128 | -1.925 | 0.055 | 0.468 |
| Cortical volume lh-rostralmiddlefrontal | 3267 | -0.015 | -0.245 | 0.806 | 0.951 |
| Cortical volume lh-superiorfrontal | 3267 | -0.020 | -0.355 | 0.723 | 0.943 |
| Cortical volume lh-superiorparietal | 3267 | 0.069 | 1.072 | 0.284 | 0.837 |
| Cortical volume lh-superiortemporal | 3267 | -0.034 | -0.532 | 0.595 | 0.865 |
| Cortical volume lh-supramarginal | 3267 | 0.036 | 0.534 | 0.593 | 0.865 |
| Cortical volume lh-frontalpole | 3267 | -0.083 | -1.164 | 0.245 | 0.758 |
| Cortical volume lh-temporalpole | 3267 | -0.056 | -0.750 | 0.454 | 0.837 |
| Cortical volume lh-transversetemporal | 3267 | 0.003 | 0.042 | 0.966 | 0.981 |
| Cortical volume lh-insula | 3267 | 0.001 | 0.011 | 0.991 | 0.991 |
| Cortical volume rh-Banks of Superior Temporal Sulcus | 3267 | 0.046 | 0.626 | 0.531 | 0.849 |
| Cortical volume rh-caudalanteriorcingulate | 3267 | 0.093 | 1.211 | 0.227 | 0.758 |
| Cortical volume rh-caudalmiddlefrontal | 3267 | -0.036 | -0.508 | 0.612 | 0.867 |
| Cortical volume rh-cuneus | 3267 | -0.024 | -0.321 | 0.749 | 0.943 |
| Cortical volume rh-entorhinal | 3267 | -0.042 | -0.528 | 0.598 | 0.865 |
| Cortical volume rh-fusiform | 3267 | -0.105 | -1.726 | 0.085 | 0.515 |
| Cortical volume rh-inferiorparietal | 3267 | -0.004 | -0.058 | 0.954 | 0.981 |
| Cortical volume rh-inferiortemporal | 3267 | 0.047 | 0.757 | 0.450 | 0.837 |
| Cortical volume rh-isthmuscingulate | 3267 | -0.057 | -0.805 | 0.421 | 0.837 |
| Cortical volume rh-lateraloccipital | 3267 | 0.011 | 0.170 | 0.865 | 0.951 |
| Cortical volume rh-lateralorbitofrontal | 3267 | -0.107 | -1.862 | 0.063 | 0.479 |
| Cortical volume rh-lingual | 3267 | -0.099 | -1.376 | 0.170 | 0.679 |
| Cortical volume rh-medialorbitofrontal | 3267 | -0.053 | -0.856 | 0.393 | 0.837 |
| Cortical volume rh-middletemporal | 3267 | 0.017 | 0.302 | 0.763 | 0.943 |
| Cortical volume rh-parahippocampal | 3267 | -0.052 | -0.708 | 0.480 | 0.837 |
| Cortical volume rh-paracentral | 3267 | 0.087 | 1.193 | 0.234 | 0.758 |
| Cortical volume rh-parsopercularis | 3267 | -0.118 | -1.583 | 0.114 | 0.599 |
| Cortical volume rh-parsorbitalis | 3267 | -0.176 | -2.565 | 0.011 | 0.366 |
| Cortical volume rh-parstriangularis | 3267 | -0.004 | -0.052 | 0.959 | 0.981 |
| Cortical volume rh-pericalcarine | 3267 | -0.043 | -0.577 | 0.564 | 0.865 |
| Cortical volume rh-postcentral | 3267 | -0.082 | -1.257 | 0.210 | 0.758 |
| Cortical volume rh-posteriorcingulate | 3267 | 0.014 | 0.198 | 0.843 | 0.951 |
| Cortical volume rh-precentral | 3267 | 0.045 | 0.710 | 0.478 | 0.837 |
| Cortical volume rh-precuneus | 3267 | 0.121 | 2.062 | 0.040 | 0.389 |
| Cortical volume rh-rostralanteriorcingulate | 3267 | -0.023 | -0.319 | 0.750 | 0.943 |
| Cortical volume rh-rostralmiddlefrontal | 3267 | -0.074 | -1.167 | 0.244 | 0.758 |
| Cortical volume rh-superiorfrontal | 3267 | -0.010 | -0.168 | 0.867 | 0.951 |
| Cortical volume rh-superiorparietal | 3267 | 0.091 | 1.403 | 0.162 | 0.679 |
| Cortical volume rh-superiortemporal | 3267 | 0.041 | 0.663 | 0.508 | 0.849 |
| Cortical volume rh-supramarginal | 3267 | -0.043 | -0.628 | 0.531 | 0.849 |
| Cortical volume rh-frontalpole | 3267 | -0.075 | -1.039 | 0.299 | 0.837 |
| Cortical volume rh-temporalpole | 3267 | -0.200 | -2.604 | 0.010 | 0.366 |
| Cortical volume rh-transversetemporal | 3267 | 0.026 | 0.359 | 0.720 | 0.943 |
| Cortical volume rh-insula | 3267 | 0.052 | 0.855 | 0.393 | 0.837 |

All models were fitted adjusted for paternal age less than 20 years at birth, race, sex, age at outcome assessment, parental income level, family environment score, T1 image signal-to-noise, and intracranial volume.

# Table S8. Associations between adolescent motherhood and cortical volumes in female offspring.

| **Outcome** | **N** | **β** | **t** | **P** | **FDR** |
| --- | --- | --- | --- | --- | --- |
| Cortical volume lh-Banks of Superior Temporal Sulcus | 3037 | -0.096 | -1.503 | 0.134 | 0.454 |
| Cortical volume lh-caudalanteriorcingulate | 3037 | -0.150 | -2.204 | 0.028 | 0.256 |
| Cortical volume lh-caudalmiddlefrontal | 3037 | -0.073 | -1.162 | 0.246 | 0.619 |
| Cortical volume lh-cuneus | 3037 | 0.012 | 0.182 | 0.856 | 0.882 |
| Cortical volume lh-entorhinal | 3037 | 0.028 | 0.452 | 0.652 | 0.738 |
| Cortical volume lh-fusiform | 3037 | -0.027 | -0.485 | 0.628 | 0.737 |
| Cortical volume lh-inferiorparietal | 3037 | -0.042 | -0.681 | 0.496 | 0.719 |
| Cortical volume lh-inferiortemporal | 3037 | 0.032 | 0.573 | 0.567 | 0.727 |
| Cortical volume lh-isthmuscingulate | 3037 | -0.035 | -0.623 | 0.534 | 0.726 |
| Cortical volume lh-lateraloccipital | 3037 | -0.036 | -0.650 | 0.516 | 0.719 |
| Cortical volume lh-lateralorbitofrontal | 3037 | -0.078 | -1.543 | 0.124 | 0.443 |
| Cortical volume lh-lingual | 3037 | 0.000 | -0.006 | 0.996 | 0.996 |
| Cortical volume lh-medialorbitofrontal | 3037 | -0.094 | -1.758 | 0.080 | 0.387 |
| Cortical volume lh-middletemporal | 3037 | 0.016 | 0.287 | 0.774 | 0.822 |
| Cortical volume lh-parahippocampal | 3037 | -0.036 | -0.531 | 0.596 | 0.737 |
| Cortical volume lh-paracentral | 3037 | -0.060 | -0.969 | 0.333 | 0.648 |
| Cortical volume lh-parsopercularis | 3037 | -0.046 | -0.713 | 0.477 | 0.719 |
| Cortical volume lh-parsorbitalis | 3037 | -0.014 | -0.220 | 0.826 | 0.864 |
| Cortical volume lh-parstriangularis | 3037 | -0.066 | -0.993 | 0.321 | 0.648 |
| Cortical volume lh-pericalcarine | 3037 | 0.040 | 0.578 | 0.564 | 0.727 |
| Cortical volume lh-postcentral | 3037 | -0.145 | -2.574 | 0.010 | 0.146 |
| Cortical volume lh-posteriorcingulate | 3037 | -0.049 | -0.819 | 0.413 | 0.703 |
| Cortical volume lh-precentral | 3037 | -0.094 | -1.718 | 0.087 | 0.387 |
| Cortical volume lh-precuneus | 3037 | -0.117 | -2.219 | 0.027 | 0.256 |
| Cortical volume lh-rostralanteriorcingulate | 3037 | -0.197 | -3.189 | 0.002 | 0.106 |
| Cortical volume lh-rostralmiddlefrontal | 3037 | 0.038 | 0.674 | 0.501 | 0.719 |
| Cortical volume lh-superiorfrontal | 3037 | -0.048 | -0.904 | 0.367 | 0.656 |
| Cortical volume lh-superiorparietal | 3037 | -0.079 | -1.325 | 0.186 | 0.542 |
| Cortical volume lh-superiortemporal | 3037 | -0.023 | -0.413 | 0.680 | 0.746 |
| Cortical volume lh-supramarginal | 3037 | -0.038 | -0.647 | 0.518 | 0.719 |
| Cortical volume lh-frontalpole | 3037 | -0.061 | -0.884 | 0.377 | 0.658 |
| Cortical volume lh-temporalpole | 3037 | -0.007 | -0.111 | 0.912 | 0.925 |
| Cortical volume lh-transversetemporal | 3037 | -0.106 | -1.638 | 0.102 | 0.387 |
| Cortical volume lh-insula | 3037 | -0.114 | -2.118 | 0.035 | 0.256 |
| Cortical volume rh-Banks of Superior Temporal Sulcus | 3037 | -0.079 | -1.287 | 0.199 | 0.542 |
| Cortical volume rh-caudalanteriorcingulate | 3037 | -0.041 | -0.602 | 0.547 | 0.727 |
| Cortical volume rh-caudalmiddlefrontal | 3037 | -0.128 | -2.047 | 0.041 | 0.256 |
| Cortical volume rh-cuneus | 3037 | -0.067 | -1.062 | 0.289 | 0.634 |
| Cortical volume rh-entorhinal | 3037 | -0.042 | -0.649 | 0.517 | 0.719 |
| Cortical volume rh-fusiform | 3037 | -0.152 | -2.876 | 0.004 | 0.146 |
| Cortical volume rh-inferiorparietal | 3037 | -0.071 | -1.231 | 0.219 | 0.573 |
| Cortical volume rh-inferiortemporal | 3037 | -0.051 | -0.919 | 0.359 | 0.656 |
| Cortical volume rh-isthmuscingulate | 3037 | -0.119 | -1.970 | 0.050 | 0.282 |
| Cortical volume rh-lateraloccipital | 3037 | -0.055 | -1.007 | 0.315 | 0.648 |
| Cortical volume rh-lateralorbitofrontal | 3037 | -0.102 | -2.056 | 0.041 | 0.256 |
| Cortical volume rh-lingual | 3037 | 0.060 | 0.949 | 0.343 | 0.648 |
| Cortical volume rh-medialorbitofrontal | 3037 | -0.152 | -2.687 | 0.008 | 0.146 |
| Cortical volume rh-middletemporal | 3037 | 0.086 | 1.667 | 0.097 | 0.387 |
| Cortical volume rh-parahippocampal | 3037 | -0.064 | -0.950 | 0.343 | 0.648 |
| Cortical volume rh-paracentral | 3037 | -0.047 | -0.758 | 0.449 | 0.719 |
| Cortical volume rh-parsopercularis | 3037 | -0.093 | -1.451 | 0.148 | 0.478 |
| Cortical volume rh-parsorbitalis | 3037 | -0.028 | -0.433 | 0.665 | 0.741 |
| Cortical volume rh-parstriangularis | 3037 | 0.072 | 1.098 | 0.273 | 0.630 |
| Cortical volume rh-pericalcarine | 3037 | 0.031 | 0.454 | 0.650 | 0.738 |
| Cortical volume rh-postcentral | 3037 | -0.037 | -0.652 | 0.515 | 0.719 |
| Cortical volume rh-posteriorcingulate | 3037 | -0.129 | -2.170 | 0.031 | 0.256 |
| Cortical volume rh-precentral | 3037 | -0.074 | -1.286 | 0.199 | 0.542 |
| Cortical volume rh-precuneus | 3037 | -0.135 | -2.566 | 0.011 | 0.146 |
| Cortical volume rh-rostralanteriorcingulate | 3037 | -0.032 | -0.498 | 0.619 | 0.737 |
| Cortical volume rh-rostralmiddlefrontal | 3037 | 0.031 | 0.513 | 0.608 | 0.737 |
| Cortical volume rh-superiorfrontal | 3037 | -0.069 | -1.295 | 0.196 | 0.542 |
| Cortical volume rh-superiorparietal | 3037 | -0.042 | -0.700 | 0.484 | 0.719 |
| Cortical volume rh-superiortemporal | 3037 | -0.019 | -0.350 | 0.727 | 0.785 |
| Cortical volume rh-supramarginal | 3037 | -0.102 | -1.716 | 0.087 | 0.387 |
| Cortical volume rh-frontalpole | 3037 | -0.074 | -1.087 | 0.278 | 0.630 |
| Cortical volume rh-temporalpole | 3037 | -0.110 | -1.657 | 0.098 | 0.387 |
| Cortical volume rh-transversetemporal | 3037 | -0.032 | -0.514 | 0.608 | 0.737 |
| Cortical volume rh-insula | 3037 | -0.059 | -1.094 | 0.275 | 0.630 |

All models were fitted adjusted for paternal age less than 20 years at birth, race, sex, age at outcome assessment, parental income level, family environment score, T1 image signal-to-noise, and intracranial volume.

# Table S9. Associations between adolescent motherhood and cortical thickness in male offspring.

| **Outcome** | **N** | **β** | **t** | **P** | **FDR** |
| --- | --- | --- | --- | --- | --- |
| Cortical thickness lh-Banks of Superior Temporal Sulcus | 3267 | 0.034 | 0.435 | 0.664 | 0.947 |
| Cortical thickness lh-caudalanteriorcingulate | 3267 | 0.083 | 1.084 | 0.279 | 0.947 |
| Cortical thickness lh-caudalmiddlefrontal | 3267 | 0.124 | 1.617 | 0.107 | 0.870 |
| Cortical thickness lh-cuneus | 3267 | -0.031 | -0.403 | 0.687 | 0.947 |
| Cortical thickness lh-entorhinal | 3267 | 0.028 | 0.366 | 0.715 | 0.947 |
| Cortical thickness lh-fusiform | 3267 | 0.107 | 1.382 | 0.168 | 0.870 |
| Cortical thickness lh-inferiorparietal | 3267 | 0.081 | 1.088 | 0.277 | 0.947 |
| Cortical thickness lh-inferiortemporal | 3267 | -0.005 | -0.067 | 0.947 | 0.947 |
| Cortical thickness lh-isthmuscingulate | 3267 | -0.028 | -0.358 | 0.720 | 0.947 |
| Cortical thickness lh-lateraloccipital | 3267 | 0.030 | 0.442 | 0.659 | 0.947 |
| Cortical thickness lh-lateralorbitofrontal | 3267 | 0.048 | 0.607 | 0.544 | 0.947 |
| Cortical thickness lh-lingual | 3267 | 0.008 | 0.116 | 0.908 | 0.947 |
| Cortical thickness lh-medialorbitofrontal | 3267 | -0.063 | -0.830 | 0.407 | 0.947 |
| Cortical thickness lh-middletemporal | 3267 | 0.009 | 0.120 | 0.904 | 0.947 |
| Cortical thickness lh-parahippocampal | 3267 | -0.056 | -0.721 | 0.471 | 0.947 |
| Cortical thickness lh-paracentral | 3267 | 0.166 | 2.205 | 0.028 | 0.707 |
| Cortical thickness lh-parsopercularis | 3267 | -0.033 | -0.425 | 0.671 | 0.947 |
| Cortical thickness lh-parsorbitalis | 3267 | 0.107 | 1.371 | 0.171 | 0.870 |
| Cortical thickness lh-parstriangularis | 3267 | 0.075 | 0.960 | 0.338 | 0.947 |
| Cortical thickness lh-pericalcarine | 3267 | 0.038 | 0.514 | 0.608 | 0.947 |
| Cortical thickness lh-postcentral | 3267 | -0.021 | -0.299 | 0.765 | 0.947 |
| Cortical thickness lh-posteriorcingulate | 3267 | 0.111 | 1.413 | 0.159 | 0.870 |
| Cortical thickness lh-precentral | 3267 | 0.100 | 1.346 | 0.179 | 0.870 |
| Cortical thickness lh-precuneus | 3267 | 0.060 | 0.788 | 0.431 | 0.947 |
| Cortical thickness lh-rostralanteriorcingulate | 3267 | 0.059 | 0.805 | 0.421 | 0.947 |
| Cortical thickness lh-rostralmiddlefrontal | 3267 | 0.130 | 1.732 | 0.084 | 0.870 |
| Cortical thickness lh-superiorfrontal | 3267 | 0.050 | 0.660 | 0.510 | 0.947 |
| Cortical thickness lh-superiorparietal | 3267 | 0.136 | 1.858 | 0.064 | 0.870 |
| Cortical thickness lh-superiortemporal | 3267 | -0.017 | -0.226 | 0.821 | 0.947 |
| Cortical thickness lh-supramarginal | 3267 | -0.039 | -0.535 | 0.593 | 0.947 |
| Cortical thickness lh-frontalpole | 3267 | 0.056 | 0.721 | 0.472 | 0.947 |
| Cortical thickness lh-temporalpole | 3267 | 0.036 | 0.461 | 0.645 | 0.947 |
| Cortical thickness lh-transversetemporal | 3267 | 0.031 | 0.393 | 0.695 | 0.947 |
| Cortical thickness lh-insula | 3267 | -0.040 | -0.528 | 0.598 | 0.947 |
| Cortical thickness rh-Banks of Superior Temporal Sulcus | 3267 | -0.063 | -0.799 | 0.425 | 0.947 |
| Cortical thickness rh-caudalanteriorcingulate | 3267 | 0.078 | 0.980 | 0.328 | 0.947 |
| Cortical thickness rh-caudalmiddlefrontal | 3267 | 0.033 | 0.427 | 0.670 | 0.947 |
| Cortical thickness rh-cuneus | 3267 | 0.059 | 0.802 | 0.423 | 0.947 |
| Cortical thickness rh-entorhinal | 3267 | 0.053 | 0.678 | 0.498 | 0.947 |
| Cortical thickness rh-fusiform | 3267 | 0.023 | 0.302 | 0.763 | 0.947 |
| Cortical thickness rh-inferiorparietal | 3267 | -0.024 | -0.328 | 0.743 | 0.947 |
| Cortical thickness rh-inferiortemporal | 3267 | 0.104 | 1.377 | 0.169 | 0.870 |
| Cortical thickness rh-isthmuscingulate | 3267 | -0.099 | -1.258 | 0.209 | 0.943 |
| Cortical thickness rh-lateraloccipital | 3267 | 0.015 | 0.228 | 0.820 | 0.947 |
| Cortical thickness rh-lateralorbitofrontal | 3267 | 0.092 | 1.195 | 0.233 | 0.943 |
| Cortical thickness rh-lingual | 3267 | -0.008 | -0.106 | 0.915 | 0.947 |
| Cortical thickness rh-medialorbitofrontal | 3267 | -0.012 | -0.152 | 0.879 | 0.947 |
| Cortical thickness rh-middletemporal | 3267 | 0.006 | 0.079 | 0.937 | 0.947 |
| Cortical thickness rh-parahippocampal | 3267 | -0.040 | -0.527 | 0.599 | 0.947 |
| Cortical thickness rh-paracentral | 3267 | 0.054 | 0.695 | 0.487 | 0.947 |
| Cortical thickness rh-parsopercularis | 3267 | -0.130 | -1.685 | 0.093 | 0.870 |
| Cortical thickness rh-parsorbitalis | 3267 | 0.194 | 2.540 | 0.012 | 0.707 |
| Cortical thickness rh-parstriangularis | 3267 | 0.072 | 0.925 | 0.356 | 0.947 |
| Cortical thickness rh-pericalcarine | 3267 | 0.008 | 0.102 | 0.919 | 0.947 |
| Cortical thickness rh-postcentral | 3267 | 0.008 | 0.104 | 0.917 | 0.947 |
| Cortical thickness rh-posteriorcingulate | 3267 | 0.107 | 1.372 | 0.171 | 0.870 |
| Cortical thickness rh-precentral | 3267 | -0.016 | -0.205 | 0.838 | 0.947 |
| Cortical thickness rh-precuneus | 3267 | 0.166 | 2.164 | 0.031 | 0.707 |
| Cortical thickness rh-rostralanteriorcingulate | 3267 | -0.060 | -0.758 | 0.449 | 0.947 |
| Cortical thickness rh-rostralmiddlefrontal | 3267 | 0.031 | 0.415 | 0.678 | 0.947 |
| Cortical thickness rh-superiorfrontal | 3267 | 0.089 | 1.188 | 0.236 | 0.943 |
| Cortical thickness rh-superiorparietal | 3267 | 0.124 | 1.656 | 0.099 | 0.870 |
| Cortical thickness rh-superiortemporal | 3267 | 0.020 | 0.251 | 0.802 | 0.947 |
| Cortical thickness rh-supramarginal | 3267 | 0.034 | 0.475 | 0.635 | 0.947 |
| Cortical thickness rh-frontalpole | 3267 | 0.014 | 0.176 | 0.861 | 0.947 |
| Cortical thickness rh-temporalpole | 3267 | -0.023 | -0.291 | 0.771 | 0.947 |
| Cortical thickness rh-transversetemporal | 3267 | 0.040 | 0.512 | 0.609 | 0.947 |
| Cortical thickness rh-insula | 3267 | -0.037 | -0.492 | 0.623 | 0.947 |

All models were fitted adjusted for paternal age less than 20 years at birth, race, sex, age at outcome assessment, parental income level, family environment score, and T1 image signal-to-noise.

# Table S10. Associations between adolescent motherhood and cortical thickness in female offspring.

| **Outcome** | **N** | **β** | **t** | **P** | **FDR** |
| --- | --- | --- | --- | --- | --- |
| Cortical thickness lh-Banks of Superior Temporal Sulcus | 3037 | -0.206 | -2.712 | 0.007 | 0.159 |
| Cortical thickness lh-caudalanteriorcingulate | 3037 | -0.081 | -1.078 | 0.282 | 0.799 |
| Cortical thickness lh-caudalmiddlefrontal | 3037 | -0.116 | -1.580 | 0.115 | 0.748 |
| Cortical thickness lh-cuneus | 3037 | 0.016 | 0.217 | 0.829 | 0.939 |
| Cortical thickness lh-entorhinal | 3037 | 0.002 | 0.023 | 0.982 | 1.000 |
| Cortical thickness lh-fusiform | 3037 | -0.157 | -2.117 | 0.035 | 0.397 |
| Cortical thickness lh-inferiorparietal | 3037 | -0.077 | -1.060 | 0.290 | 0.799 |
| Cortical thickness lh-inferiortemporal | 3037 | -0.022 | -0.289 | 0.773 | 0.938 |
| Cortical thickness lh-isthmuscingulate | 3037 | -0.043 | -0.553 | 0.580 | 0.877 |
| Cortical thickness lh-lateraloccipital | 3037 | -0.063 | -0.977 | 0.329 | 0.799 |
| Cortical thickness lh-lateralorbitofrontal | 3037 | -0.035 | -0.466 | 0.641 | 0.928 |
| Cortical thickness lh-lingual | 3037 | -0.084 | -1.190 | 0.235 | 0.799 |
| Cortical thickness lh-medialorbitofrontal | 3037 | -0.066 | -0.876 | 0.382 | 0.799 |
| Cortical thickness lh-middletemporal | 3037 | 0.001 | 0.015 | 0.988 | 1.000 |
| Cortical thickness lh-parahippocampal | 3037 | -0.184 | -2.485 | 0.013 | 0.229 |
| Cortical thickness lh-paracentral | 3037 | -0.027 | -0.373 | 0.710 | 0.928 |
| Cortical thickness lh-parsopercularis | 3037 | -0.002 | -0.027 | 0.978 | 1.000 |
| Cortical thickness lh-parsorbitalis | 3037 | 0.030 | 0.394 | 0.694 | 0.928 |
| Cortical thickness lh-parstriangularis | 3037 | -0.076 | -1.000 | 0.318 | 0.799 |
| Cortical thickness lh-pericalcarine | 3037 | 0.043 | 0.606 | 0.545 | 0.877 |
| Cortical thickness lh-postcentral | 3037 | -0.038 | -0.512 | 0.609 | 0.900 |
| Cortical thickness lh-posteriorcingulate | 3037 | -0.088 | -1.184 | 0.237 | 0.799 |
| Cortical thickness lh-precentral | 3037 | -0.100 | -1.342 | 0.181 | 0.799 |
| Cortical thickness lh-precuneus | 3037 | -0.008 | -0.113 | 0.910 | 0.988 |
| Cortical thickness lh-rostralanteriorcingulate | 3037 | 0.004 | 0.055 | 0.956 | 1.000 |
| Cortical thickness lh-rostralmiddlefrontal | 3037 | -0.048 | -0.656 | 0.512 | 0.871 |
| Cortical thickness lh-superiorfrontal | 3037 | -0.022 | -0.298 | 0.766 | 0.938 |
| Cortical thickness lh-superiorparietal | 3037 | 0.154 | 2.144 | 0.033 | 0.397 |
| Cortical thickness lh-superiortemporal | 3037 | 0.016 | 0.223 | 0.824 | 0.939 |
| Cortical thickness lh-supramarginal | 3037 | -0.059 | -0.804 | 0.422 | 0.799 |
| Cortical thickness lh-frontalpole | 3037 | -0.031 | -0.399 | 0.690 | 0.928 |
| Cortical thickness lh-temporalpole | 3037 | -0.048 | -0.633 | 0.527 | 0.874 |
| Cortical thickness lh-transversetemporal | 3037 | -0.065 | -0.873 | 0.383 | 0.799 |
| Cortical thickness lh-insula | 3037 | -0.078 | -1.072 | 0.284 | 0.799 |
| Cortical thickness rh-Banks of Superior Temporal Sulcus | 3037 | -0.151 | -2.037 | 0.042 | 0.412 |
| Cortical thickness rh-caudalanteriorcingulate | 3037 | 0.029 | 0.380 | 0.704 | 0.928 |
| Cortical thickness rh-caudalmiddlefrontal | 3037 | -0.070 | -0.937 | 0.350 | 0.799 |
| Cortical thickness rh-cuneus | 3037 | -0.064 | -0.893 | 0.372 | 0.799 |
| Cortical thickness rh-entorhinal | 3037 | -0.019 | -0.256 | 0.798 | 0.939 |
| Cortical thickness rh-fusiform | 3037 | -0.210 | -2.899 | 0.004 | 0.136 |
| Cortical thickness rh-inferiorparietal | 3037 | -0.040 | -0.559 | 0.577 | 0.877 |
| Cortical thickness rh-inferiortemporal | 3037 | -0.106 | -1.427 | 0.154 | 0.799 |
| Cortical thickness rh-isthmuscingulate | 3037 | -0.064 | -0.833 | 0.405 | 0.799 |
| Cortical thickness rh-lateraloccipital | 3037 | -0.071 | -1.106 | 0.270 | 0.799 |
| Cortical thickness rh-lateralorbitofrontal | 3037 | -0.078 | -1.047 | 0.296 | 0.799 |
| Cortical thickness rh-lingual | 3037 | -0.022 | -0.312 | 0.755 | 0.938 |
| Cortical thickness rh-medialorbitofrontal | 3037 | -0.054 | -0.706 | 0.481 | 0.838 |
| Cortical thickness rh-middletemporal | 3037 | 0.025 | 0.347 | 0.729 | 0.935 |
| Cortical thickness rh-parahippocampal | 3037 | -0.221 | -3.010 | 0.003 | 0.136 |
| Cortical thickness rh-paracentral | 3037 | -0.068 | -0.906 | 0.365 | 0.799 |
| Cortical thickness rh-parsopercularis | 3037 | -0.065 | -0.860 | 0.390 | 0.799 |
| Cortical thickness rh-parsorbitalis | 3037 | 0.061 | 0.782 | 0.435 | 0.799 |
| Cortical thickness rh-parstriangularis | 3037 | -0.055 | -0.717 | 0.474 | 0.838 |
| Cortical thickness rh-pericalcarine | 3037 | 0.000 | 0.000 | 1.000 | 1.000 |
| Cortical thickness rh-postcentral | 3037 | 0.015 | 0.199 | 0.842 | 0.939 |
| Cortical thickness rh-posteriorcingulate | 3037 | 0.008 | 0.107 | 0.915 | 0.988 |
| Cortical thickness rh-precentral | 3037 | -0.138 | -1.817 | 0.070 | 0.597 |
| Cortical thickness rh-precuneus | 3037 | -0.064 | -0.858 | 0.392 | 0.799 |
| Cortical thickness rh-rostralanteriorcingulate | 3037 | 0.016 | 0.222 | 0.824 | 0.939 |
| Cortical thickness rh-rostralmiddlefrontal | 3037 | -0.060 | -0.802 | 0.423 | 0.799 |
| Cortical thickness rh-superiorfrontal | 3037 | -0.074 | -0.998 | 0.319 | 0.799 |
| Cortical thickness rh-superiorparietal | 3037 | 0.040 | 0.557 | 0.578 | 0.877 |
| Cortical thickness rh-superiortemporal | 3037 | -0.097 | -1.302 | 0.194 | 0.799 |
| Cortical thickness rh-supramarginal | 3037 | -0.030 | -0.424 | 0.672 | 0.928 |
| Cortical thickness rh-frontalpole | 3037 | -0.065 | -0.846 | 0.398 | 0.799 |
| Cortical thickness rh-temporalpole | 3037 | -0.087 | -1.185 | 0.237 | 0.799 |
| Cortical thickness rh-transversetemporal | 3037 | -0.110 | -1.555 | 0.121 | 0.748 |
| Cortical thickness rh-insula | 3037 | -0.126 | -1.753 | 0.080 | 0.608 |

All models were fitted adjusted for paternal age less than 20 years at birth, race, sex, age at outcome assessment, parental income level, family environment score, and T1 image signal-to-noise.

# Table S11. Associations between adolescent motherhood and subcortical volumes in male offspring.

| **Outcome** | **N** | **β** | **t** | **P** | **FDR** |
| --- | --- | --- | --- | --- | --- |
| Volume left-cerebral-white-matter | 3267 | -0.044 | -1.126 | 0.261 | 0.912 |
| Volume left-lateral-ventricle | 3267 | 0.070 | 0.871 | 0.384 | 0.912 |
| Volume left-inf-lat-vent | 3267 | -0.012 | -0.149 | 0.881 | 1.000 |
| Volume left-cerebellum-white-matter | 3267 | -0.054 | -0.816 | 0.415 | 0.912 |
| Volume left-cerebellum-cortex | 3267 | -0.030 | -0.512 | 0.609 | 0.912 |
| Volume left-thalamus-proper | 3267 | -0.036 | -0.648 | 0.517 | 0.912 |
| Volume left-caudate | 3267 | 0.034 | 0.488 | 0.626 | 0.912 |
| Volume left-putamen | 3267 | 0.000 | 0.003 | 0.998 | 1.000 |
| Volume left-pallidum | 3267 | -0.043 | -0.622 | 0.534 | 0.912 |
| Volume 3rd-ventricle | 3267 | -0.035 | -0.453 | 0.651 | 0.912 |
| Volume 4th-ventricle | 3267 | -0.080 | -1.036 | 0.301 | 0.912 |
| Volume brain-stem | 3267 | -0.014 | -0.242 | 0.809 | 0.986 |
| Volume left-hippocampus | 3267 | -0.101 | -1.670 | 0.096 | 0.912 |
| Volume left-amygdala | 3267 | -0.043 | -0.682 | 0.496 | 0.912 |
| Volume csf | 3267 | -0.114 | -1.560 | 0.120 | 0.912 |
| Volume left-accumbens-area | 3267 | 0.046 | 0.722 | 0.471 | 0.912 |
| Volume left-ventraldc | 3267 | -0.037 | -0.708 | 0.479 | 0.912 |
| Volume right-cerebral-white-matter | 3267 | -0.047 | -1.204 | 0.230 | 0.912 |
| Volume right-lateral-ventricle | 3267 | 0.014 | 0.170 | 0.865 | 1.000 |
| Volume right-inf-lat-vent | 3267 | -0.082 | -1.046 | 0.296 | 0.912 |
| Volume right-cerebellum-white-matter | 3267 | 0.000 | 0.000 | 1.000 | 1.000 |
| Volume right-cerebellum-cortex | 3267 | -0.004 | -0.063 | 0.950 | 1.000 |
| Volume right-thalamus-proper | 3267 | 0.004 | 0.083 | 0.934 | 1.000 |
| Volume right-caudate | 3267 | 0.061 | 0.893 | 0.372 | 0.912 |
| Volume right-putamen | 3267 | -0.027 | -0.396 | 0.692 | 0.912 |
| Volume right-pallidum | 3267 | -0.064 | -0.922 | 0.357 | 0.912 |
| Volume right-hippocampus | 3267 | -0.078 | -1.232 | 0.219 | 0.912 |
| Volume right-amygdala | 3267 | -0.053 | -0.839 | 0.402 | 0.912 |
| Volume right-accumbens area | 3267 | 0.038 | 0.576 | 0.565 | 0.912 |
| Volume right-ventraldc | 3267 | -0.028 | -0.523 | 0.601 | 0.912 |
| Volume wm-hypointensities | 3267 | 0.031 | 0.391 | 0.696 | 0.912 |
| Volume CC-Posterior | 3267 | -0.058 | -0.751 | 0.453 | 0.912 |
| Volume CC-Mid_Posterior | 3267 | 0.050 | 0.650 | 0.516 | 0.912 |
| Volume CC-Central | 3267 | 0.003 | 0.031 | 0.975 | 1.000 |
| Volume CC-Mid_Anterior | 3267 | 0.030 | 0.384 | 0.701 | 0.912 |
| Volume CC-Anterior | 3267 | 0.020 | 0.264 | 0.792 | 0.986 |
| Volume wholebrain | 3267 | -0.031 | -1.109 | 0.268 | 0.912 |
| Volume latventricles | 3267 | 0.043 | 0.540 | 0.590 | 0.912 |
| Volume allventricles | 3267 | 0.032 | 0.406 | 0.685 | 0.912 |

All models were fitted adjusted for paternal age less than 20 years at birth, race, sex, age at outcome assessment, parental income level, family environment score, T1 image signal-to-noise, and intracranial volume.

# Table S12. Associations between adolescent motherhood and subcortical volumes in female offspring.

| **Outcome** | **N** | **β** | **t** | **P** | **FDR** |
| --- | --- | --- | --- | --- | --- |
| Volume left-cerebral-white-matter | 3037 | 0.030 | 0.881 | 0.379 | 0.778 |
| Volume left-lateral-ventricle | 3037 | 0.078 | 1.250 | 0.212 | 0.752 |
| Volume left-inf-lat-vent | 3037 | -0.013 | -0.190 | 0.849 | 0.987 |
| Volume left-cerebellum-white-matter | 3037 | 0.001 | 0.016 | 0.987 | 0.987 |
| **Volume left-cerebellum-cortex** | **3037** | **-0.161** | **-3.194** | **0.002** | **0.060** |
| Volume left-thalamus-proper | 3037 | 0.008 | 0.177 | 0.860 | 0.987 |
| Volume left-caudate | 3037 | -0.036 | -0.599 | 0.549 | 0.931 |
| Volume left-putamen | 3037 | -0.002 | -0.031 | 0.975 | 0.987 |
| Volume left-pallidum | 3037 | 0.026 | 0.458 | 0.648 | 0.987 |
| Volume 3rd-ventricle | 3037 | 0.023 | 0.366 | 0.715 | 0.987 |
| Volume 4th-ventricle | 3037 | -0.070 | -0.999 | 0.319 | 0.778 |
| Volume brain-stem | 3037 | -0.050 | -0.986 | 0.325 | 0.778 |
| Volume left-hippocampus | 3037 | -0.013 | -0.245 | 0.807 | 0.987 |
| Volume left-amygdala | 3037 | -0.091 | -1.614 | 0.107 | 0.680 |
| Volume csf | 3037 | -0.010 | -0.149 | 0.882 | 0.987 |
| Volume left-accumbens-area | 3037 | -0.083 | -1.359 | 0.175 | 0.683 |
| Volume left-ventraldc | 3037 | 0.006 | 0.126 | 0.899 | 0.987 |
| Volume right-cerebral-white-matter | 3037 | 0.037 | 1.077 | 0.282 | 0.778 |
| Volume right-lateral-ventricle | 3037 | 0.103 | 1.702 | 0.090 | 0.680 |
| Volume right-inf-lat-vent | 3037 | 0.188 | 2.597 | 0.010 | 0.128 |
| Volume right-cerebellum-white-matter | 3037 | -0.054 | -0.886 | 0.376 | 0.778 |
| Volume right-cerebellum-cortex | 3037 | -0.135 | -2.685 | 0.008 | 0.128 |
| Volume right-thalamus-proper | 3037 | -0.022 | -0.460 | 0.646 | 0.987 |
| Volume right-caudate | 3037 | -0.067 | -1.129 | 0.260 | 0.778 |
| Volume right-putamen | 3037 | -0.038 | -0.637 | 0.525 | 0.930 |
| Volume right-pallidum | 3037 | 0.041 | 0.731 | 0.466 | 0.865 |
| Volume right-hippocampus | 3037 | -0.007 | -0.122 | 0.903 | 0.987 |
| Volume right-amygdala | 3037 | 0.016 | 0.288 | 0.774 | 0.987 |
| Volume right-accumbens area | 3037 | -0.093 | -1.451 | 0.148 | 0.680 |
| Volume right-ventraldc | 3037 | 0.037 | 0.786 | 0.433 | 0.843 |
| Volume wm-hypointensities | 3037 | -0.008 | -0.112 | 0.911 | 0.987 |
| Volume CC-Posterior | 3037 | 0.003 | 0.043 | 0.966 | 0.987 |
| Volume CC-Mid_Posterior | 3037 | 0.076 | 1.090 | 0.277 | 0.778 |
| Volume CC-Central | 3037 | -0.016 | -0.239 | 0.811 | 0.987 |
| Volume CC-Mid_Anterior | 3037 | -0.064 | -0.911 | 0.363 | 0.778 |
| Volume CC-Anterior | 3037 | 0.013 | 0.199 | 0.842 | 0.987 |
| Volume wholebrain | 3037 | -0.037 | -1.533 | 0.126 | 0.680 |
| Volume latventricles | 3037 | 0.098 | 1.593 | 0.112 | 0.680 |
| Volume allventricles | 3037 | 0.087 | 1.419 | 0.157 | 0.680 |

All models were fitted adjusted for paternal age less than 20 years at birth, race, sex, age at outcome assessment, parental income level, family environment score, T1 image signal-to-noise, and intracranial volume.

# Table S13. Associations between brain morphology that were associated with adolescent motherhood and neurodevelopmental outcomes.

| **Exposure** | **Outcome** | **Sex** | **N** | **Adjusted β, 95% CI^*^** |
| --- | --- | --- | --- | --- |
| Cortical area of pars orbitalis | NIH Toolbox | Male | 3215 | 0.55 (0.22 to 0.87) |
| Volume of left cerebellum cortex | NIH Toolbox | Female | 2976 | 1.47 (1.14 to 1.80) |
| **Exposure** | **Outcome** | **Sex** | **N** | **Adjusted mean ratio, 95% CI^*^** |
| Cortical area of pars orbitalis | CBCL externalizing | Male | 3266 | 0.93 (0.89 to 0.97) |
| Volume of left cerebellum cortex | CBCL internalizing | Female | 3037 | 1.00 (0.97 to 1.04) |
| Volume of left cerebellum cortex | SSRS | Female | 2614 | 0.92 (0.87 to 0.97) |

SSRS, Short-Social Responsiveness Scale; CBCL, Child Behavioral Checklist.

**^*^** Adjusted for paternal age less than 20 years at birth, race, sex, age at outcome assessment, parental income level, and family environment.

# Table S14. Associations between adolescent motherhood and brain morphology in two randomly selected subgroups at baseline and 2-year follow-up.

| **Outcome** | **Sex** | **Follow-up** | **Group** | **N** | **Adjusted β, 95% CI^*^** | **P** |
| --- | --- | --- | --- | --- | --- | --- |
| Cortical area of pars orbitalis, left | Male | Baseline | 1 | 1447 | -0.42 (-0.65 to -0.19) | <0.001 |
| Cortical area of pars orbitalis, left | Male | Baseline | 2 | 1820 | -0.14 (-0.33 to 0.04) | 0.13 |
| Cortical area of pars orbitalis, right | Male | Baseline | 1 | 1447 | -0.33 (-0.57 to -0.09) | 0.007 |
| Cortical area of pars orbitalis, right | Male | Baseline | 2 | 1820 | -0.29 (-0.47 to -0.11) | 0.002 |
| Volume of left cerebellum cortex | Female | Baseline | 1 | 1353 | -0.14 (-0.33 to 0.06) | 0.16 |
| Volume of left cerebellum cortex | Female | Baseline | 2 | 1684 | -0.14 (-0.29 to 0.02) | 0.08 |
| Cortical area of pars orbitalis, left | Male | 2-year follow-up | 1 | 988 | -0.55 (-0.85 to -0.25) | <0.001 |
| Cortical area of pars orbitalis, left | Male | 2-year follow-up | 2 | 1273 | -0.21 (-0.42 to 0.01) | 0.06 |
| Cortical area of pars orbitalis, right | Male | 2-year follow-up | 1 | 988 | -0.36 (-0.67 to -0.05) | 0.03 |
| Cortical area of pars orbitalis, right | Male | 2-year follow-up | 2 | 1273 | -0.32 (-0.53 to -0.10) | 0.005 |
| Volume of left cerebellum cortex | Female | 2-year follow-up | 1 | 839 | -0.15 (-0.41 to 0.10) | 0.24 |
| Volume of left cerebellum cortex | Female | 2-year follow-up | 2 | 1178 | -0.10 (-0.28 to 0.08) | 0.28 |

**^*^** Adjusted for paternal age less than 20 years at birth, race, sex, age at outcome assessment, parental income level, and family environment.

# Additional Methods S1. Model selection for outcomes.

The Short-Social Responsiveness Scale (SSRS), the Child Behavioral Checklist (CBCL) internalizing, and CBCL externalizing score were three heavily right-skewed outcomes. The original and log-transformed scores are presented below (Appendix figure 1). We observed that the distribution of log-transformed SSRS and CBCL externalizing scores were still skewed. We tried three strategies to model the data: linear regression on the raw score, linear regression on the log-transformed score, and Poisson regression on the raw score. Overdispersion test showed no sign of overdispersion of all the three scores, thus we did not further apply a negative-binomial regression. The right-hand side of the model included maternal age, sex, an interaction term of age × sex, family income, race, age at assessment, adolescent father at birth, and a random factor of family nested within sites. We compared the normalized mean squared error (NMSE) as an indicator of goodness-of-fit. NMSE was calculated by:

$$NMSE=\frac{1}{N}\left( y-\hat{y} \right)^{2}/Var(y)$$

where N denotes the sample size, y denotes the outcome, $\hat{y}$ denotes the predicted outcome, and Var(y) denotes the variance of y. NMSE permits comparison between different scales of outcomes (in our case, raw scale and log-transformed scale), where lower value indicate better model fit. We found that Poisson regression on the raw score showed lower NMSE. Thus, we applied Poisson regression in our following analyses.


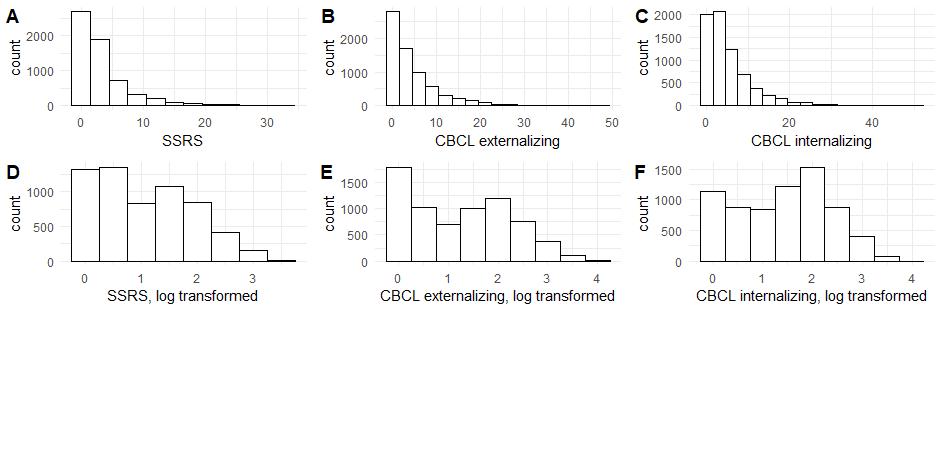


**Appendix figure 1. Distribution of SSRS, CBCL externalizing, CBCL internalizing scores, and their log-transformed scores, respectively, in the study population.**

**Appendix table 1. Goodness-of-fit of three right-skewed outcomes, modeled by different strategies.**

| **Outcome** | **Modeling** | **NMSE** |
| --- | --- | --- |
| **SSRS** | Raw, linear | 0.55 |
| **SSRS** | Log-transformed, linear | 0.45 |
| **SSRS** | Raw, Poisson | 0.05 |
| **CBCL externalizing** | Raw, linear | 0.36 |
| **CBCL externalizing** | Log-transformed, linear | 0.33 |
| **CBCL externalizing** | Raw, Poisson | 0.03 |
| **CBCL internalizing** | Raw, linear | 0.28 |
| **CBCL internalizing** | Log-transformed, linear | 0.33 |
| **CBCL internalizing** | Raw, Poisson | 0.03 |
